# A droplet microfluidic platform for high-throughput photochemical reaction discovery

**DOI:** 10.1038/s41467-020-19926-z

**Published:** 2020-12-03

**Authors:** Alexandra C. Sun, Daniel J. Steyer, Anthony R. Allen, Emory M. Payne, Robert T. Kennedy, Corey R. J. Stephenson

**Affiliations:** Department of Chemistry, University of Michigan, Ann Arbor, MI USA

**Keywords:** Automation, Flow chemistry, Photocatalysis

## Abstract

The implementation of continuous flow technology is critical towards enhancing the application of photochemical reactions for industrial process development. However, there are significant time and resource constraints associated with translating discovery scale vial-based batch reactions to continuous flow scale-up conditions. Herein we report the development of a droplet microfluidic platform, which enables high-throughput reaction discovery in flow to generate pharmaceutically relevant compound libraries. This platform allows for enhanced material efficiency, as reactions can be performed on picomole scale. Furthermore, high-throughput data collection via on-line ESI mass spectrometry facilitates the rapid analysis of individual, nanoliter-sized reaction droplets at acquisition rates of 0.3 samples/s. We envision this high-throughput screening platform to expand upon the robust capabilities and impact of photochemical reactions in drug discovery and development.

## Abstract (web summary)

Translating discovery scale vial-based batch reactions to continuous flow scale-up conditions is limited by significant time and resource constraints. Here, the authors report a photochemical droplet microfluidic platform, which enables high throughput reaction discovery in flow to generate pharmaceutically relevant compound libraries.

## Introduction

High-throughput experimentation (HTE) techniques hold the potential to revolutionize modern catalysis and reaction discovery by enabling the exploration of myriad reaction conditions in a time and resource-efficient manner^1–6^. In recent years, efforts have been directed towards the development of mass spectrometry-based (MS) HTE systems for the automated processing of Pd-based cross-coupling reactions on nanomole scale, in both batch and continuous flow settings^7–10^. In particular, the development of flow-based HTE platforms enables the direct optimization of continuous flow reactions in a material-efficient and data-rich manner. Researchers at Pfizer have developed a modular, automated system to enable nanomole scale reaction discovery in-flow at a throughput of over 1500 samples/day (0.02 samples/s)^9^. While reported HTE flow systems have been successfully applied to non-photochemical reactions, demand remains for the development of HTE platforms for screening photochemical reactions in continuous flow.

Among the repertoire of modern catalytic methods, photoredox catalysis has enabled otherwise infeasible bond disconnections and aided sustainability efforts in industrial synthesis^11^. The widespread implementation of photoredox catalysis renders the development and adaptation of flow technology to be broadly impactful for reaction scale-up^12,13^. The greater surface area-to-volume ratio accessible in-flow results in increased photon flux, which can lead to reaction acceleration^12^. Translating discovery-scale batch conditions to pilot-scale flow operations often necessitates significant resource consumption. Additionally, the process of re-optimization for flow conditions can prove laborious and cost-inefficient^14^. While commercially available systems have been designed for photochemical high-throughput batch (i.e., microvial-based) experimentation^15^, there is comparatively limited capability for performing flow-based screening of photochemical reactions. Those flow-based screening systems that have been reported lack the throughput of their batch counterparts^16–18^. With the end goal of developing scalable photochemical flow reactions, we aimed to build an HTE system to enable reaction discovery in-flow.

We have identified droplet microfluidics as a platform that may enable continuous flow-based reaction discovery. Segmentation of samples with an immiscible phase enables the simultaneous handling of numerous samples over extended periods of time^19–21^. From a material consumption standpoint, droplet microfluidics screens are typically performed at nanoliter to femtoliter scale, which translates to a reduction in starting material usage by three to eight orders of magnitude relative to a traditional multiwell plate-based screen. Droplet microfluidics is also well suited for photocatalytic reactions, as the micrometer dimension of the reaction vessel allows for high photon flux through the reaction channel in an analogous manner to the narrow tubing employed in-flow reactors^22–24^. Here, we report advances towards a droplet microfluidic/MS platform to enable picomole-scale discovery of visible light-driven reactions and provide robust translatability to millimole flow scale-up processes. In particular, the design of a flow-based screening platform that interfaces with pre-plated compound libraries would allow for facile integration into existing pharmaceutical HTE workflows and infrastructures. We foresee the synergistic combination of droplet microfluidics, MS, and photoredox catalysis as an enabling platform for accelerating pharmaceutical discovery and development, with a concerted emphasis on time and material efficiency.

## Results

### ESI-MS analysis of in-droplet reactions

**Fig. 1:**
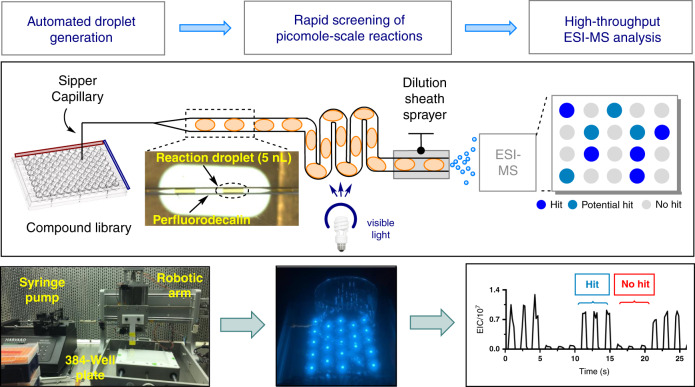
Development of a photochemical droplet microfluidics platform. EIC extracted ion chromatogram, ESI-MS electrospray ionization-mass spectrometry.

**Fig. 2:**
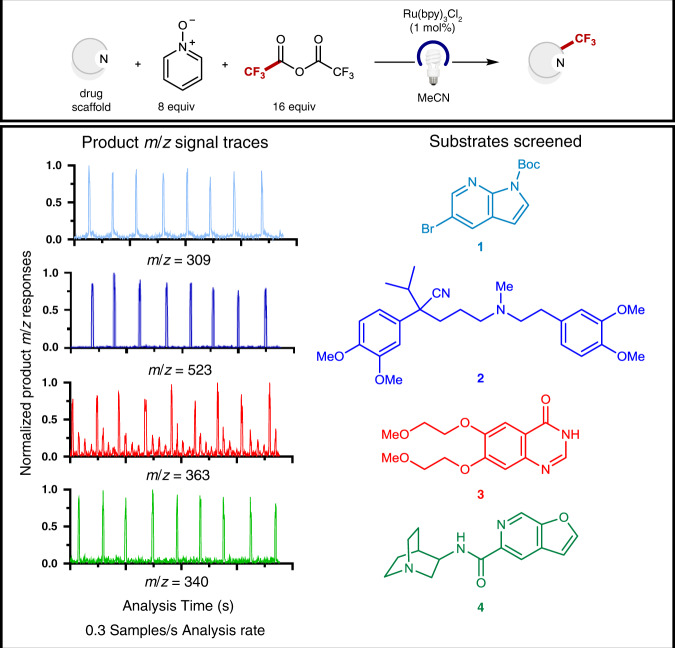
ESI-MS analysis of in-droplet trifluoromethylation reactions. MH^+^ molecular ions were monitored for all trifluoromethylated products except for 1, which was prominently observed as a *m*/*z* = 309 fragment. Ru(bpy)_3_Cl_2_ tris(bipyridine)ruthenium(II) chloride.

In this work, we aimed to develop a system for irradiating droplet samples, followed by in-line dilution and subsequent electrospray ionization-mass spectrometry (ESI-MS) analysis (Fig. 1). Droplet samples (5–10 nL) were generated from a standard 384 or 1536 microwell plate using previously reported methods^25–28^, enabling the facile translation of a wide variety of reaction conditions into droplet format. In our preliminary studies, we constructed an easily assembled, low-footprint photoreactor (see Supplementary Information for details). Following droplet irradiation, simultaneous dilution and ESI-MS analysis of each individual droplet sample was performed using a sheath sprayer, which enables in-line dilution of samples as they are pumped into the ESI source^25^. Dilution of droplets served to both quench the reaction and facilitate MS analysis, as the analysis of high concentration (>1 mM) analytes can lead to saturation of MS signal and contamination of the MS source. In our preliminary studies, we chose to employ a radical trifluoromethylation developed by the Stephenson group^29^ as a model reactive system for the late-stage functionalization of complex pharmaceutical intermediates. To demonstrate the general operation of our system, we performed in-droplet trifluoromethylation of four distinct substrates (Fig. 2, 1–4), three of which were either approved therapeutics or drug candidates provided by a commercial library from Pfizer^30^. Reaction droplets (4 nL), segmented by a perfluorodecalin (PFD) carrier phase (8 nL), were formed in a 100 µm internal diameter (i.d.) PFA tubing. Droplet reactions containing different substrates were generated in a consecutive manner. After a 10 min irradiation, droplets flowed at 500 nL/min into the sheath sprayer for subsequent dilution. ESI-MS analysis of droplet samples was performed at 0.3 samples/s. As anticipated, prominent product *m*/*z* signals were observed iteratively throughout the four traces in the expected alternating pattern. These results validate the capability to perform rapid MS analysis while maintaining the identity of individual samples. Notably, the analytes were not subject to Taylor dispersion as in a conventional plug flow reactor^12^. For the reactions in Fig. 2, we also observed reproducible results in both our droplet-to-droplet analysis and in-droplet chemistries. (Supplementary Fig. 3). Droplet ESI-MS measurement of reaction turnover yielded RSD values ranging from 1–19% across all four reactions, both for reactions run prior to droplet generation and for reactions performed inside of droplets. In this manner, we established proof-of-concept for the successful performance and ESI-MS analysis of in-droplet organic synthesis reactions.

### In-droplet reaction discovery

In the next phase of our studies, we set out to investigate in-droplet photoredox flow reactions. To perform residence time optimization, we designed an oscillatory flow system to enable extended irradiation of reaction droplets (Supplementary Fig. 5). Oscillation was induced by alternating a syringe pump between withdrawal and infusion modes while maintaining a constant flow rate of 200 nL/min. Initial validation of our oscillatory system was performed using a visible light-driven alkene aminoarylation reaction^31^. Successful product formation was observed by ESI-MS analysis, with droplet identities maintained after a 1 h residence time. Notably, while state-of-the-art oscillatory flow systems have been limited to a single reaction plug during each incubation period, this setup could potentially accommodate >100× more samples per incubation period^19,32^.

**Fig. 3:**
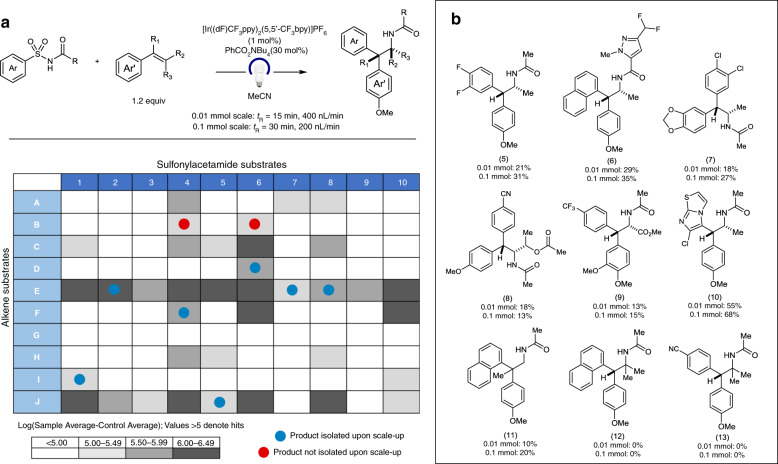
Droplet microfluidics-enabled HTE reaction discovery on the picomole scale. **a** Heatmap of coupling 10 sulfonylacetamides with a matrix of 10 alkene substrates. Gray boxes denote potential hit/hit responses. Boxes containing red and blue circles indicate reactions that were performed on 0.01 mmol and 0.1 mmol scale. **b** Product yields for millimole scale-up flow reactions. [Ir((dF)CF_3_ppy)_2_(5,5′-CF_3_bpy)]PF_6_ 4,4′-di-*tert*-butyl-2,2′-bipyridine-bis-3,5-difluoro-2-[5-(trifluoromethyl)-2-phenylpyridine] iridium (III) hexafluorophosphate.

After observing success with our droplet flow reactor, we sought to perform HTE reaction discovery to generate a library of alkene aminoarylation products. We aimed to screen an extensive library of sulfonylacetamides and alkenes on a picomole scale to furnish a wealth of reactivity data generated from each substrate combination. Ten sulfonylacetamides and ten alkenes were selected for our screen (500 pmol scale), resulting in the potential generation of 100 distinct product combinations (Fig. 3). For these studies, droplet reactions were irradiated using a custom-built Cree LED array photoreactor, in order to maximize photon flux^27^. Our oscillatory flow setup was employed to maintain a residence time of 30 min at a flow rate of 200 nL/min during reaction irradiation (100–200 reaction droplets per incubation period). ESI-MS analysis was performed at a throughput of 0.3 samples/s (350 total samples over 19 min). ESI-MS results suggested the identification of 37 hit conditions, for which significant product *m*/*z* signals were observed. The employment of trans-anethole yielded product formation across the entire scope of sulfonylacetamide substrates, in-line with reported studies in batch^31^. Notably, pharmaceutically relevant sulfonylacetamides containing heteroarenes (6,10) also yielded several hit responses in our droplet screen. Furthermore, this data suggests that electron deficient sulfonylacetamides generally gave rise to enhanced reactivity and broader alkene compatibility. Our in-droplet reaction discovery screen has significantly expanded upon the scope of our reported alkene aminoarylation methodology to incorporate substrates of increased structural complexity, as well as enabled the elucidation of reactivity trends and structure-activity relationships to inform ongoing mechanistic studies.

### Continuous flow scale-up validation

To validate our ESI-MS screen results, we selected nine of our droplet reactions to perform 0.01 mmol scale-up in-flow and enable subsequent product isolation (Fig. 3). These reactions were carried out in the same reactor (100 µm i.d., 360 o.d.) and run in a continuous stream (non-droplet format) at a flow rate of 400 nL/min, providing a residence time of 15 min. Upon irradiation, purification was performed using mass-directed HPLC methods. Of the nine reactions selected, seven of the hit reactions were successfully validated through product isolation. Product isolation was unsuccessful in the case of compounds 12 and 13. Of the selected reactions, these yielded the weakest product MS signals in our original droplet reaction screen and had been categorized as potential hits. Control experiments suggest the formation of an unidentified byproduct that gives the same *m*/*z* signal as the desired product (see Supplementary Information for details). Finally, we set out to demonstrate the translatability of our droplet screen results to a microscale flow reaction, in order to generate milligram scale quantities of material for discovery chemistry applications. Our 0.1 mmol scale reactions were irradiated in a 100 µL volume PFA reactor (0.03″ i.d.) with a residence time of 30 min at a flow rate of 3 µL/min. The same set of nine reactions were scaled up to yield isolation results that showed a strong correlation with that of the 0.01 mmol scale flow reactions. These experiments highlight the utility of our droplet microfluidics platform for enabling reaction discovery in a high-throughput, material efficient, and data-rich manner. At the same time, this platform shows significant promise in its translatability and applicability towards discovery chemistry scale-up to facilitate library synthesis for initial biological studies.

### Design of a reagent addition system

**Fig. 4:**
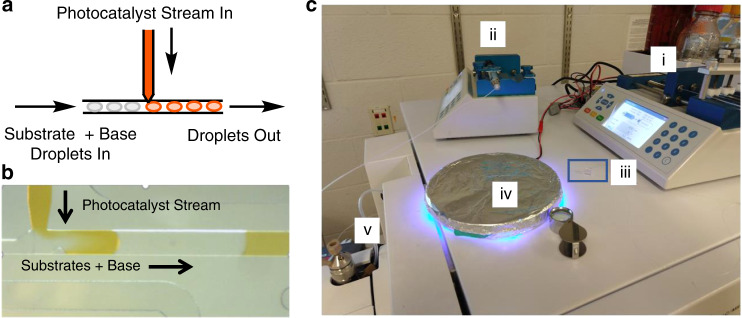
Incorporation of a microfluidic chip for reagent addition. **a** Reagent addition device design. **b** Device in operation. Each incoming droplet from the left received a solution from the upper channel and moved right to export. **c** Full setup for in-droplet flow reaction screening. (i) Syringe pump driving both droplet flow and reagent flow into reagent addition chip, (ii) Syringe pump driving sheath flow, (iii, in blue box) Reagent addition device, (iv) Photoreactor chamber, (v) Sheath sprayer for ESI-MS analysis.

While the droplets used in the reactors shown in Figs. 2 and 3 contain nanoliters of reagents, the true reagent consumption was microliters that were deposited into the well plate. To fully realize the potential for reduced reagent consumption, we integrated automated reagent addition capabilities into a droplet reactor (Fig. 4a). By adding reagents (e.g., photocatalyst) directly to substrate-containing droplets, reagent consumption can be reduced to picomoles per reaction. As depicted in Fig. 4, preformed substrate-containing acetonitrile droplets were flowed from PFA tubing into a reagent addition device, through an addition region to receive photocatalyst, and exported to PFA tubing for irradiation and ESI-MS analysis (throughput of 0.3 samples/s) in one continuous system. Consistent volume addition (<9% relative standard deviation for added reagent in two separate experiments; 48 droplets examined in each experiment) and low droplet-to-droplet carry-over were observed in preliminary experiments (Supplementary Fig. 8). This platform was subsequently utilized to perform and monitor in-droplet photochemistry. ESI-MS analysis of aminoarylation droplets after photocatalyst addition and irradiation (7 min residence time) showed consistent product formation across all droplet samples (Supplementary Fig. 9). Because these reactions are performed continuously, after the initial 7 min lag, reactions are analyzed at a rate limited by detection, i.e., 0.3 samples/s. These experiments provide proof-of-concept for the enhanced material efficiency of our developed platform, as droplet reactions were prepared and analyzed at <1% sample usage relative to conventional µL-scale screens while providing high-throughput in a flow-based reaction. We envision the applicability of this system towards screening efforts in photocatalysis, as well as other non-photochemical catalytic transformations, in which reagents are expensive or limited in supply.

## Discussion

The droplet microfluidics system described in this study provides a high-throughput continuous flow platform for screening large volumes of photochemical reactions in a miniaturized fashion. The use of droplet microfluidics presents several significant advantages for performing in-flow reaction discovery. Our oscillatory in-droplet screening setup (Fig. 3) allows for the simultaneous irradiation of up to 100 picomole-scale reaction droplets, representing a marked improvement from state-of-the-art systems that typically accommodate one sample/incubation period (Supplementary Table 1)^19,32^. Furthermore, this system enables in-droplet reaction discovery to rapidly generate high-volume compound libraries and provide access to greater magnitudes of chemical space. ESI-MS analysis of reaction droplets was performed at a throughput of 0.3 samples/s, which represents a marked improvement over other ESI-MS systems for the analysis of flow reactions^20^. Upon translating our picomole-scale droplet reactions to millimole scale flow conditions, we have also validated the successful flow scale-up of our droplet reactions to enable product isolation.

In comparison with plate-based screening approaches^7,8,10^, our system provides enhanced levels of material efficiency (500 pmol of material usage per reaction vs. 1–50 nmol) and high acquisition throughputs (Supplementary Table 1). While DESI-MS (desorption electrospray ionization-mass spectrometry) methods provide superior analytical throughputs (1–2 samples/s), they do not accommodate photochemical flow reactions, as reaction samples need to be analyzed in plate format. Compared to other similar screening platforms^9,20,24^, the platform reported here expands upon the utility of flow-based HTE systems beyond Suzuki cross-coupling and thermal reactions to enable screening of photochemical conditions, shows significantly enhanced material efficiency (500 pmol per reaction vs. 200–800 nmol) and increases analytical throughput over 10-fold (0.3 samples/s vs. 0.02 samples/s).

Future studies will be targeted towards developing a microfluidic chip-based system to enable in-line droplet generation, reagent addition, incubation, and MS analysis. Automated data processing capabilities will be also be developed to accommodate the wealth of HTE data generated from larger screening campaigns. From an early discovery standpoint, this droplet microfluidics HTE platform will facilitate rapid photochemical reaction discovery in-flow for the expedited generation of compound libraries. We anticipate that droplet microfluidics-driven HTE will continue to expand and enhance the utility of photochemical transformations from the bench to the drug pipeline.

## Methods

### Procedure for trifluoromethylation in-droplet reactions

Photocatalyst (1 mol%), pyridine *N*-oxide (4 equiv.), and acetonitrile (0.2 M) were added to a vial charged with a stir bar. The solution was sparged with a stream of nitrogen gas for 5 min. Trifluoroacetic anhydride (4 equiv.) was subsequently added, and the solution was stirred for 10 min to facilitate the formation of the acylated species. Separate solutions of the substrate in acetonitrile (0.2 M) were also prepared. 10 µL of each solution were combined in a PCR tube to yield the final reaction mixture, which was then deposited into a 1536 multiwell plate for droplet generation. Droplets of 4 nL volume were formed from each mixture into reactor tubing (100 µm i.d., 360 µm o.d.) and placed into a reactor dish (Supplementary Fig. 2) for irradiation. After 10 min of irradiation, droplet samples were characterized by ESI-MS analysis.

### Representative procedure for Smiles–Truce rearrangement in-droplet reaction screen

To a flame dried 1-dram vial was added tetrabutylammonium benzoate (30 mol%), and [Ir(dF(CF_3_)ppy)_2_(5,5’d(CF_3_)bpy)]PF_6_ photocatalyst (1 mol%). The vial contents were then dissolved in anhydrous acetonitrile (0.2 M). Finally, the alkene was added (1.2 equiv.). This solution was sparged under argon for 15 min. Separate solutions of the substrate in acetonitrile (0.2 M) were also prepared. For reactions formed directly from well-plates, 10 µL of each solution were deposited into a well to form the final reaction mixture. Three droplets were made for every reaction condition, with the average droplet response reported. 10 droplets for every sulfonamide substrate were run in which no alkene reagent was present to generate an average control value. Following droplet generation, reactor tubing (100 µm i.d., 360 µm o.d.) containing droplets was wrapped around a 100 × 50 mm glass recrystallization dish and placed on top of our 25 LED array light setup (Supplementary Fig. 4) for irradiation. Droplet reactions were run at a flow rate of 200 nL/min in an oscillatory manner by programming a syringe pump to alternate between refill and infusion modes at 10 min intervals, yielding a total residence time of 1 h. It is noteworthy that residence times were varied based on experiment. Following irradiation, droplet samples were characterized by ESI-MS analysis.

### ESI-MS analysis of droplet samples

Tubing containing droplets was threaded through capillary electrophoresis (CE) ESI-MS sprayer until ~0.5 mm was protruding. Sheath and droplet flows were driven by Fusion 400 syringe pumps. Droplets were flowed into the sheath sprayer (Supplementary Fig. 2) and merged with a dilution stream of 50:50 methanol:water w/0.5% formic acid (100 µL/min flow rate). ESI-MS analysis was performed on an Agilent 6410 triple quadrupole mass spectrometer. ESI potential was set to 2500 V, nebulizer gas to 15 psi, and drying gas from MS source flowed at 10 L/min at 325 °C. The mass spectrometer was set to scan from 75 to 750 *m*/*z* at 73 ms per scan. Droplet responses for any given *m*/*z* value were taken as the average of three consecutive data points from within each droplet’s observed peak.

## Supplementary information

Supplementary Information

## Data Availability

The data that support the findings of this study are available from the corresponding author upon reasonable request.
