# Supplementary material for: A droplet microfluidic platform for high-throughput photochemical reaction discovery
Source: Nat Commun. 2020 Dec 3;11:6202. doi: 10.1038/s41467-020-19926-z (PMC7712835; doi:10.1038/s41467-020-19926-z)
Supplement: Supplementary file 1 — Supplementary Information [file 41467_2020_19926_MOESM1_ESM.pdf]

Supplementary Information for

A Droplet Microfluidic Platform for High-Throughput Photochemical Reaction Discovery

Alexandra C. Sun,[†‡] Daniel J. Steyer,[†‡] Anthony R. Allen,[†] Emory M. Payne,[†] and Robert T. Kennedy,[*†] Corey R. J. Stephenson.[*†]

Correspondence to: crjsteph@umich.edu, rtkenn@umich.edu

This PDF file includes:

Materials and Methods

Figures S1 to S9

References 1-11

Table of Contents

Materials and Instrumentation 3
Droplet Generation Method 4
ESI-MS Analysis of Droplet Samples 4
In-Droplet Photoredox Trifluoromethylation Reactions 5
In-Droplet Photoredox Smiles-Truce Rearrangement Reactions 7
Reagent Addition System 10
Benchmarking Analysis of Reaction Screening Platforms 13
Compound Characterization 14
References 30

Materials and Instrumentation

Chemicals were either used as received or purified according to the procedures outlined in *Purification of Common Laboratory Chemicals*. Perfluorodecalin (PFD) and trichloro(1*H*,1*H*,2*H*,2*H*-perfluorooctyl)silane were purchased from Oakwood Products (Estill, SC). All other reagents were purchased from Fisher Scientific or Sigma Aldrich. Hygroscopic *N*-oxide substrates were dried on a high vacuum line for 6 h at ambient temperature prior to use. Pyridine *N*-oxide was dried on a high vacuum line at 60 °C for 12 hours. Thin-layer chromatography (TLC) analysis of reaction mixtures was performed using Merck silica gel 60 F254 TLC plates and visualized by a dual short wave/long wave UV lamp. Column flash chromatography was performed using 230–400 mesh silica gel or automatically by a Biotage Isolera Four, with Biotage SNAP KP-Sil 25 or 50 g flash chromatography cartridges. Mass-directed purification was performed with an Agilent high performance liquid chromatography/mass spectrometry (HPLC/MS) auto purification system using electrospray ionization (ESI), positive ion mode.

ESI-MS analysis of droplet samples was performed on an Agilent 6410 triple quadrupole mass spectrometer (Agilent Technologies, Santa Clara, CA). Nuclear magnetic resonance (NMR) spectra were recorded using an internal deuterium lock on Varian MR400, Varian Inova 500 and Varian VNMRs 700 spectrometers. Chemical shifts for ^{1}HNMR were reported as δ, parts per million, relative to the signal of $CHCl_3$ at 7.26 ppm. Chemical shifts for ^{13}CNMR were reported as δ, parts per million, relative to the center line signal of the $CDCl_3$ triplet at 77.36 ppm. Multiplicities are reported using the following abbreviations: s = singlet, d = doublet, t = triplet, q = quartet, quint = quintet, m = multiplet, br = broad resonance, dd = doublet of doublet, dt = doublet of triplet, etc. High-resolution mass spectra (HRMS) for compound characterization were recorded at the Mass Spectrometry Facility at the Department of Chemistry of the University of Michigan in Ann Arbor, MI, on a Micromass AutoSpec Ultima Magnetic Sector mass spectrometer using ESI, positive ion mode. IR spectra were recorded on a Perkin-Elmer Spectrum BX FT-IR spectrometer fitted with an ATR accessory.

LED light strips (used in our in-droplet trifluoromethylation reactions) and the requisite power box and cables were purchased from Creative Lighting Solutions (http://www.creativelightings.com) with the following item codes: CL-FRS5050-12WP-12V (4.4 W blue LED light strip), CL-PS94670-25W (25 W power supply), CL-PC6FT-PCW (power cord), CL-TERMBL-5P (terminal block). LED and heat sink parts for our 25 Cree LED array photoreactor were purchased from LED Supply (https://ledsupply.com) with the following item codes: CREEXTE-ROY-X (XLamp XT-E Royal Blue LEDs), MAKERSLED (MakersLED Heat Sink), PDA060B-XXXB (60W Phihong IP67 Constant Current AC Drivers). A Westpointe Electrical Co Wp 4'' Hi Velocity Fan 1002 Personal Fan was purchased from https://amazon.com.

Droplet Generation Method

Droplet generation from microwell plates (MWPs) was performed using equipment and methods described previously (*1*,2). Samples were drawn into either 100 µm inner diameter (i.d) x 360 µm outer diameter (o.d.) perfluoroalkoxyalkane (PFA) tubing (IDEX Health and Science, Oak Harbor, WA) by a PHD 2000 Programmable syringe pump (Harvard Apparatus, Holliston, TX). A 25 µL glass syringe (Hamilton, 1700 Series Gastight Syringe, N Termination, Part No. 80285) filled with PFD and connected to a reducing union, 1/16'' to 360 µm (Valco Instruments Co., Houston, TX, Part No. C360TU1PK6) and ferrule (Valco Instruments Co., Houston TX, Part No. C360NFPK) was mounted onto the syringe pump. 6 µL samples were deposited into 1536 polymerase chain reaction (PCR) MWPs (Corning, Corning, NY) covered with a layer of PFD. While solution was being withdrawn through the tubing, an XYZ-position manipulator moved the tubing between sample wells and fluorous phase to form alternating droplet/carrier phase trains.

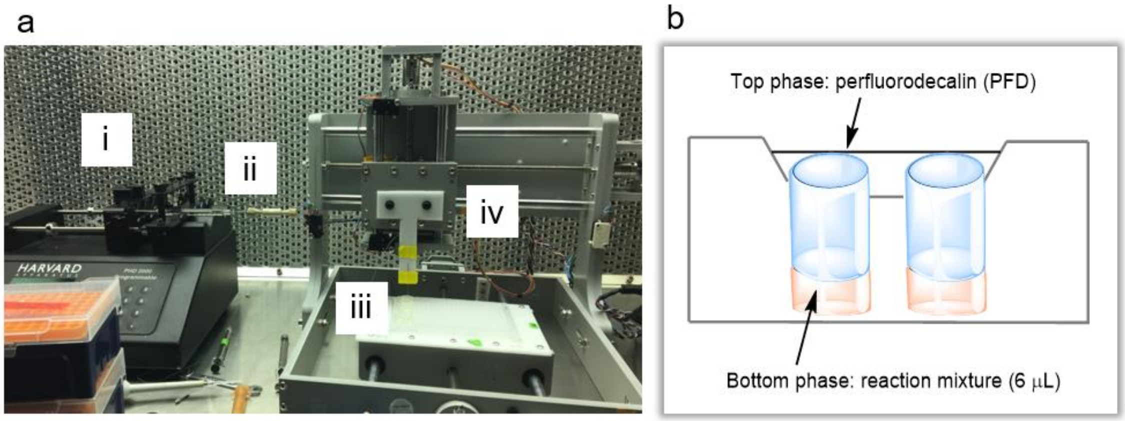

Supplementary Figure 1. Setup for automated droplet generation. (a) Generation of droplets from 1536 well plates with (i) syringe pump, (ii) syringe, (iii) 1536 microwell plate, (iv) XYZ-position manipulator with well plate stage. (b) Zoomed in depiction of wells containing reaction mixture and PFD.

ESI-MS Analysis of Droplet Samples

Tubing containing droplets was threaded through a capillary electrophoresis (CE) ESI-MS sprayer (Agilent Technologies, Santa Clara, CA) until approximately 0.5 mm was protruding. Sheath and droplet flows were driven by Fusion 400 syringe pumps (Chemyx, Stafford, TX). Droplets were flowed into the sheath sprayer (Supplementary Figure 2) and merged with a dilution stream of 50:50 methanol:water w/ 0.5% formic acid (100 µL/min flow rate). ESI-MS analysis was performed on an Agilent 6410 triple quadrupole mass spectrometer (Agilent Technologies, Santa Clara, CA). ESI potential was set to 2500 V, nebulizer gas to 15 psi, and drying gas from MS source was flowed at 10 L/min at 325 ºC. Mass spectrometer was set to scan from 75 to 750 m/z at 73 ms per scan. Droplet responses for any given m/z value were taken as the average of 3 consecutive data points from within each droplet's observed peak.

In-Droplet Photoredox Trifluoromethylation Reactions

LED Light Strip Photoreactor Setup

This photoreactor setup was used for the photoredox trifluoromethylation experiments described in Figure 2 of the main text and Supplementary Figures 3 and 9 of the SI. A 150 mm wide x 15 mm deep polystyrene petri dish was lined with aluminum foil to promote internal reflection of light. A 4.4 W blue LED strip (Creative Lighting Solutions, Columbia Station, OH) was placed around the edge of the interior of the dish (Supplementary Figure 2). A small slit was cut from the petri dish wall to run wires and tubing through. A small hole was cut out of the reactor wall to allow for the LED power cord and droplet tubing to enter. A coil of perfluoroalkoxy (PFA) tubing (100 µm inner diameter; 360 µm outer diameter) was coiled around the center of the reactor 2 cm from the light strip.

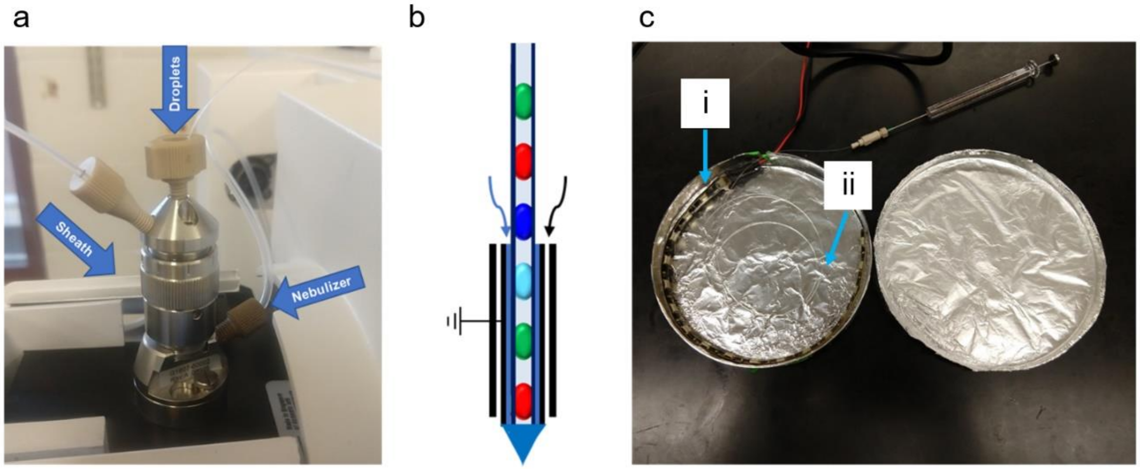

Supplementary Figure 2. Setup for irradiation and ESI-MS analysis of droplet samples. (a) Setup of sheath sprayer. (b) Schematic of sheath sprayer for droplet work. Tubing with droplets runs through the middle of the sprayer. Sheath liquid flows directly around tubing (Blue arrow). Electrospray is aided by use of nebulizer gas (Black arrow) (c) Photoreactor setup. To perform in-tubing droplet reactions, a petri dish was coated with aluminum foil, with an LED array (i) lining the rim and reactor PFA tubing (ii) coiled at the center.

General Procedure A: Preparation of Trifluoromethylation Reaction Solutions

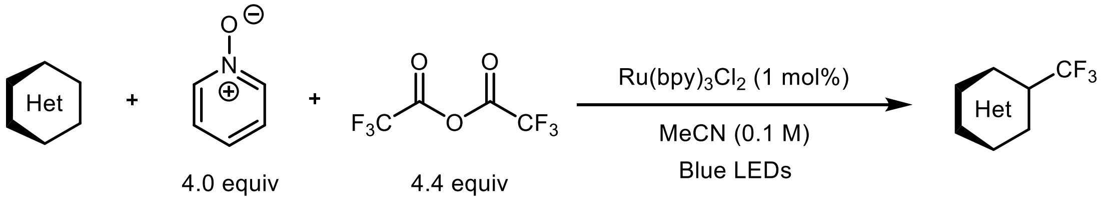

Photocatalyst (1 mol%), pyridine N-oxide (4 equiv), and acetonitrile (0.2 M) were added to a vial charged with a stir bar. The solution was sparged with a stream of nitrogen gas for 5 min. Acetic anhydride (4 equiv) was subsequently added, and the solution was stirred for 10 min to facilitate formation of the acylated species. Separate solutions of substrate in acetonitrile (0.2 M) were also prepared. 10 µL of each solution were combined in a PCR tube to yield the final reaction mixture.

Procedure for In-Droplet and Non-Droplet Control Experiments

Experiments were conducted to compare reactivity in 4 nL droplets with batch reactions run on a standard multiwell plate screen scale of 20 µL (Supplementary Figure 3). 4 nL droplet reactions were prepared and run according to the aforementioned procedures and irradiated for 10 min inside of our photoreactor. 20 µL reactions were performed in PCR tubes, with the PCR tubes placed directly in the middle of the photoreactor for 10 min of irradiation. Following irradiation, the solutions were then formed into 4 nL droplets for direct comparison to the two volume scales. The quotient of the product signal over the summed product (P) and substrate (S) signals ($\frac{P}{P+S}$) was used to appraise reaction progress. For the two substrates that performed the best in the 20 µL reactions (1 and 2), only slight increases in product formation were observed when run in droplet format; however, the increase was drastic for the lower performing substrates (3 and 4) (Supplementary Figure 3). The changes in reaction performance can be attributed to the narrower sample geometry. The 100 µm i.d. tubing presents a substantially narrower pathlength, lowering the amount of light absorbed and possibly promoting more uniform irradiation across the entire sample. Such an effect could be helpful in promoting the observation of product in poorly performing reactions, or in reducing reaction time requirements in screening for both flow reaction and batch reaction screening.

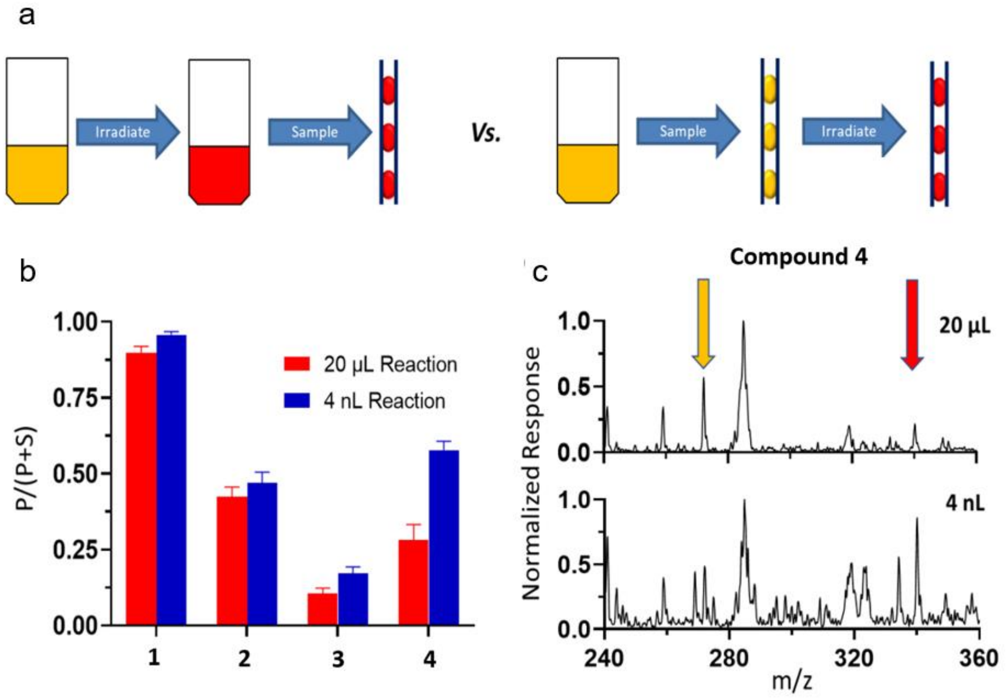

Supplementary Figure 3. Comparison of in-droplet reactions vs. non-droplet batch reactions. (a) General schemes for running reactions at different scales (Left) Reactions run at 20 µL were irradiated immediately after mixing in PCR tubes and then reformatted into 4 nL droplets for analysis (Right) For in-droplet reactions, premixed solution was reformatted into 4 nL droplets, which were then irradiated. (b) Evaluation of performance across 4 substrates in either 20 µL or 4 nL volume. In every case, P/(P+S) response was found to be similar or significantly higher in droplet format. N=20 droplets for each reaction. (c) Example spectra from both 20 µL (Top) or 4 nL (Bottom) volume PF15 reactions. The yellow arrow indicates substrate m/z value, while the red arrow indicates product m/z value. In the 20 µL reaction, the substrate response was over double that of the product; however, the product response was even greater than that of the substrate in the 4 nL reaction.

In-Droplet Photoredox Smiles-Truce Rearrangement Reactions

25 LED Array Photoreactor Setup

This photoreactor setup was used for the photoredox Smiles-Truce Rearrangement experiments described in Figure 3 of the main text and Supplementary Figures 4 and 7. A 25 LED array of Cree Royal Blue XTE LEDs (2 W per LED, 50 W total output) was assembled (Supplementary Figure 4). The LEDs were mounted onto a heat sink, with two fans placed below and adjacent to the heat sink, in order to provide sufficient cooling to maintain reactions at ambient temperatures. An acrylic shield positioned 5 cm above the LED array provided a mounting stage for the reactor tubing, as well as an additional layer of protection for the LEDs. A custom-built plastic amber light shield (built by Ann Arbor Plastics, Saline, MI) was placed around the setup for user eye protection.

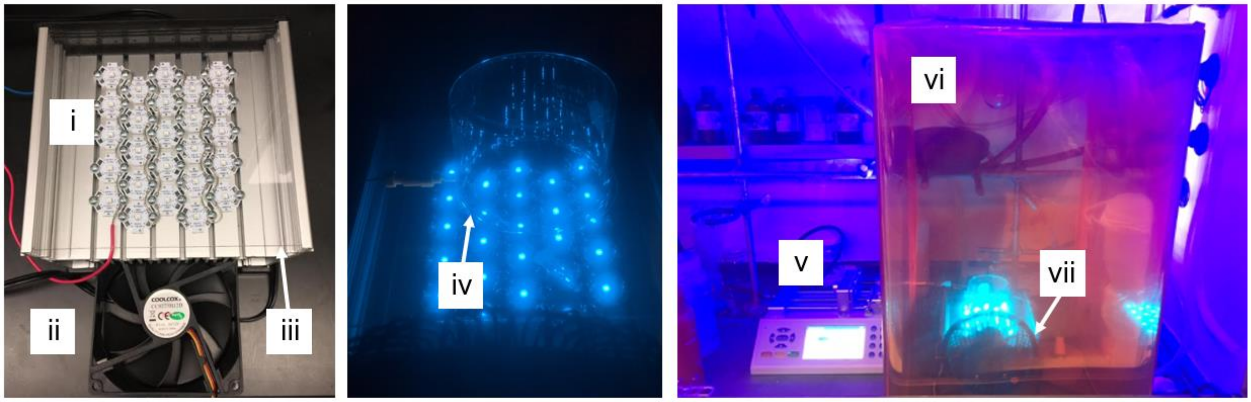

Supplementary Figure 4. (Left) Cree Royal Blue XT-E LED photoreactor setup with (i) 25 array of Cree LEDs, (ii) fan positioned underneath heat sink, (iii) acrylic shield. (Middle) Close-up view of irradiation device in operation with (iv) PFA reactor tubing containing reaction droplets. (Right) Entire setup for irradiation of in-droplet Smiles-Truce rearrangement reactions with (v) syringe pump, (vi) amber protective shield, (vii) fan positioned adjacent to heat sink.

General Procedure B: Preparation of Smiles-Truce Rearrangement Reaction Solutions

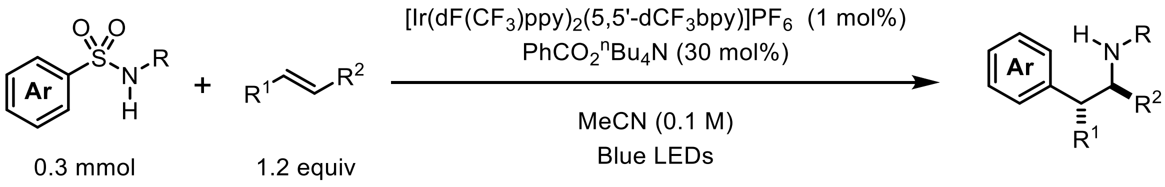

To a flame dried 1-dram vial was added tetrabutylammonium benzoate (30 mol%), and $[Ir(dF(CF_3)ppy)_2(5,5'd(CF_3)bpy)]PF_6$ photocatalyst (1 mol%). The vial contents were then dissolved in anhydrous acetonitrile (0.2 M). Finally, the alkene was added (1.2 equiv). This solution was sparged under argon for 15 min. Separate solutions of substrate in acetonitrile (0.2 M) were also prepared. For reactions formed directly from well-plates, 10 µL of each solution were deposited into a well to form the final reaction mixture. Three droplets were made for every reaction condition, with the average droplet response reported. 10 droplets for every sulfonamide substrate were run in which no alkene reagent was present to generate an average control value.

Droplet reactions were irradiated based on the method outlined in General Procedure C (see below).

Oscillating Flow Reactor

To allow for extended reaction times in-flow inside of our PFA tubing, an oscillatory flow scheme was employed (Supplementary Figure 5). We implemented a visible light-driven alkene aminoarylation reaction as our system of choice to provide us with a manifold for performing high throughput reaction discovery in continuous flow. Droplets were formed from substrate and reaction mixture into a 100 µm i.d. PFA tube. In Supplementary Figure 5, droplet contents alternating between containing the N-((3,4-difluorophenyl)sulfonyl)acetamide substrate and the N-((4-cyanophenyl)sulfonyl)acetamide substrate, denoted as 3,4-F and 4-CN sulfonylacetamides respectively. A PCR tube had a 400 µm hole drilled in the cap and was filled with PFD. The outlet of the tubing was threaded through the hole and submerged in PFD to avoid evaporation of samples inside of the tubing. Upon irradiation, the droplets were flowed at 200 nL/min, first withdrawing towards the syringe pump for 10 min, followed by 10 min of infusing away from the syringe. This process was performed 3 times, allowing for 1 hour of continuous flow reaction. Upon analysis under the same conditions as the previous experiments, both reactions were observable in alternating fashion by monitoring the product m/z traces. Measured turnover for both reactions was found to be significant by Product signal/(Product signal + Substrate Signal), with values of 0.919 ± 0.022 for the 4-CN product and 0.499 ± 0.044 for the 3,4-F product in the droplet samples shown in Supplementary Figure S5 (product traces shown).

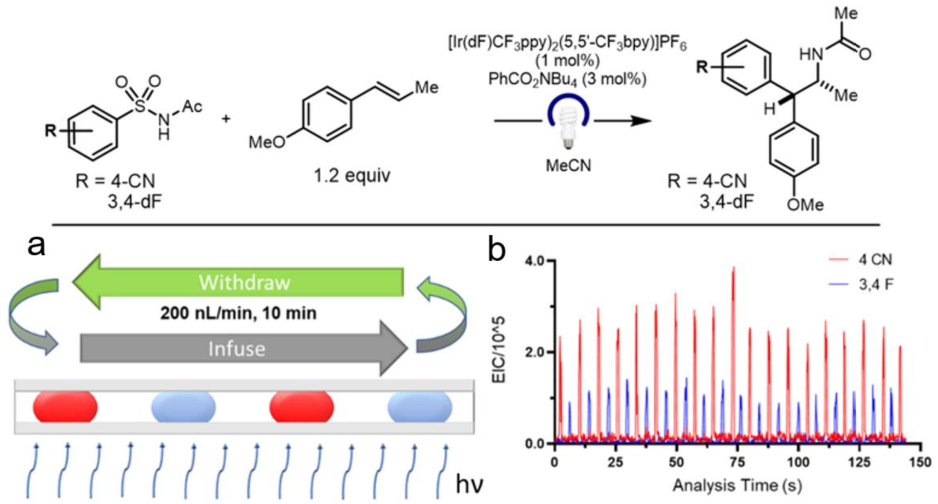

Supplementary Figure 5. Reaction of droplets in oscillating flow. (a) Scheme for oscillating flow reactor. While being irradiated, droplet flow was cycled between moving towards (withdraw) or away from (infuse) the syringe to allow for continuous flow in a linear, volume limited reactor. (b) Extracted traces for 4 CN (m/z = 309) and 3,4 F (m/z = 320) substrate reaction products, showing the formation of the two products in alternating droplets.

The current setup allowed for simultaneous irradiation of 40 reaction droplets and could conceivably increase to >100 droplets with longer tubing lengths and shorter oscillation periods. As currently demonstrated systems for oscillating flow in a reactor have been limited to a single plug, such a setup would amount to over 100x more samples to be irradiated in a single incubation period (*3*).

General Procedure C: In-Droplet Reaction Screen Setup

Following droplet generation, reactor tubing (100 µm i.d., 360 µm o.d.) containing droplets was wrapped around a 100 x 50 mm glass recrystallization dish and placed on top of our 25 LED array light setup (Supplementary Figure 4) for irradiation. Droplet reactions were run at a flow rate of 200 nL/min in an oscillatory manner by programming a syringe pump to alternate between refill and infusion modes at 10 min intervals, yielding a total residence time of 1 h. Following irradiation, droplet samples were characterized by ESI-MS analysis. Upon ESI-MS analysis, product m/z signals from each reaction were compared to the same m/z signal from the controls (blank droplets) to generate the heatmap in Figure 3. A minimum increase of 10^5 in extracted ion count (EIC) was set as the threshold for a hit, as it represented the lowest increase required to observe droplet signal over background noise. For reagent addition experiments, droplets were formed from substrate solution, and reagents were added on-chip.

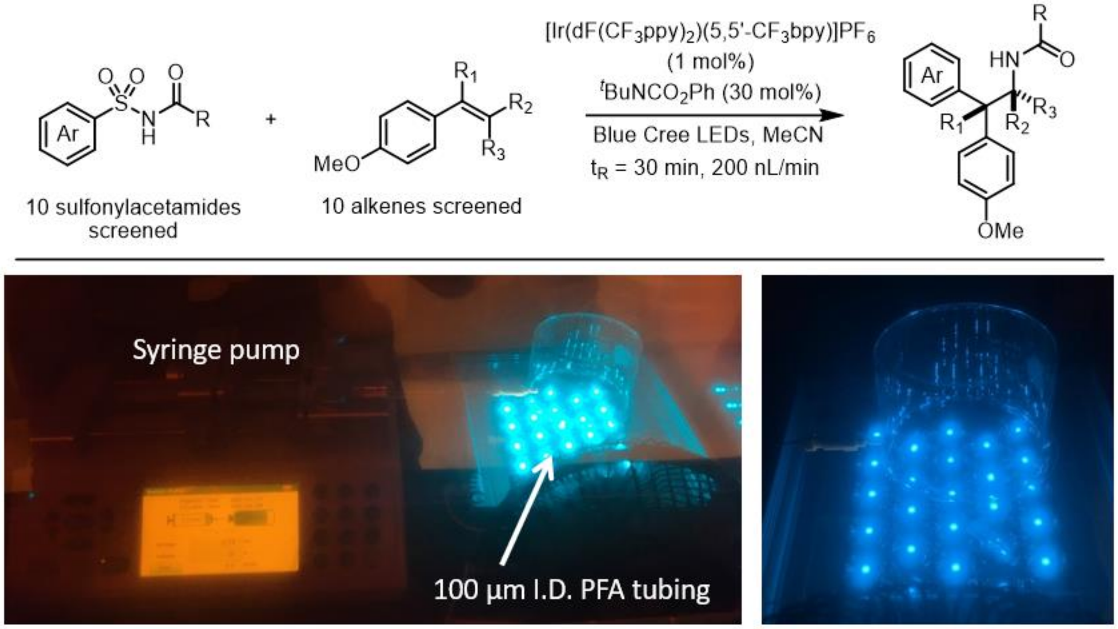

Supplementary Figure 6. Continuous flow setup for in-droplet reaction screens.

General Procedure D: Flow Scale-up (0.01 mmol) Setup

We selected 9 of our droplet reactions to perform 0.01 mmol scale-up in flow to provide sufficient material for product isolation (see Figure 3). Reactions were set up according to General Procedure B. These reactions were carried out in the same PFA tubing (100 um i.d., 360 o.d.) and run in a continuous stream (non-droplet format) at a flow rate of 400 nL/min, providing a residence time of 15 min. Irradiation was performed using the 25 LED array setup described in Chapter 3. Purification was performed using an HPLC-MS (Agilent) with an eluent of 50% MeCN/ H_2O (50% to 100% MeCN/ H_2O across 30 min).

General Procedure E: Flow Scale-up (0.1 mmol) Setup

We selected 9 of our droplet reactions to perform 0.1 mmol scale-up in flow (see Figure 3). Reactions were set up according to General Procedure B. These reactions were carried out in PFA tubing (0.03'' i.d., 1/16'' o.d., 100 µL internal volume) and run in a continuous stream (non-droplet format) at a flow rate of 300 nL/min, providing a residence time of 30 min. Irradiation was

performed using the 25 LED array setup described above. Purification was performed using flash chromatography on SiO_2 with an eluent of 70% ethyl acetate (spiked with 1% acetic acid)/hexanes (20% to 70% ethyl acetate/hexanes gradient across 20 min).

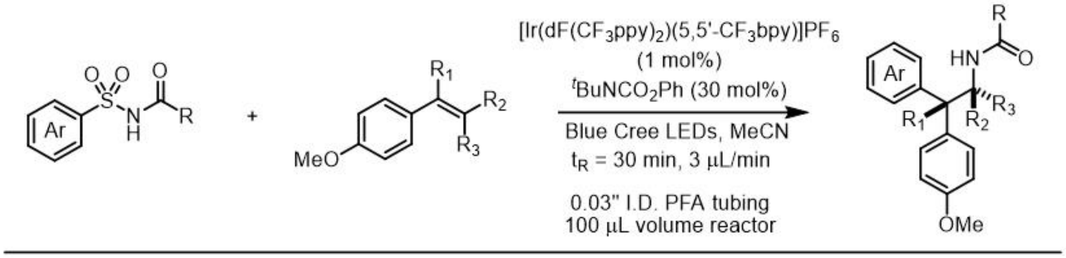

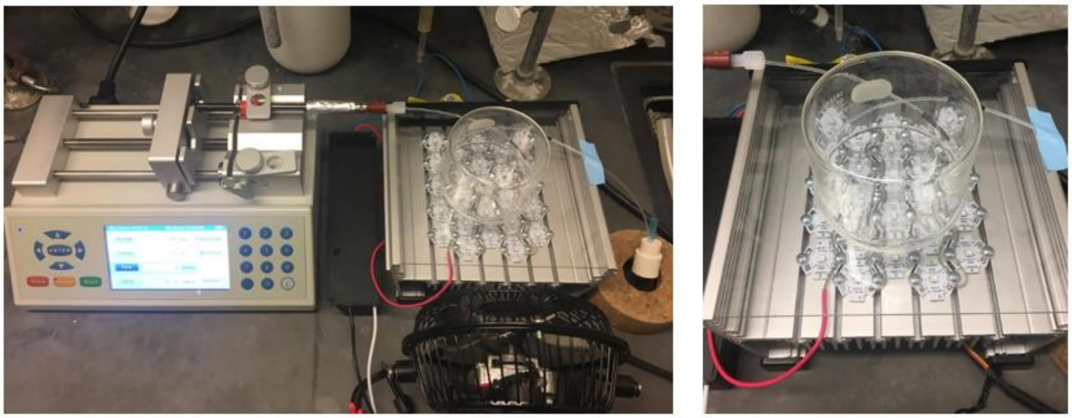

Supplementary Figure 7. Continuous flow setup for 0.1 mmol scale up reactions.

Reagent Addition System

Chip Fabrication

Microfluidic chips were fabricated using standard soft lithography procedures (*4*,*5*). SU-8 2050 photoresist was spun to 100 µm depth on silicon wafers (University Wafer, Boston, MA) then developed using photolithography to form negative masters. Uncured PDMS (Curbell Plastics, Livonia, MI) was poured on top of clean masters or blank wafers and allowed to cure for 1 h at 65 °C. Patterned PDMS and blank PDMS were baked for 1 h at 150 °C, followed by 1 min of exposure to atmospheric plasma and baking for 2 h at 150 °C to create an irreversible bond. Chip channel surfaces were treated with 2% trichloro(1H,1H,2H,2H-perfluorooctyl)silane in PFD by flowing 10 µL internal volumes through over 10 min followed by 2 h of baking at 65 ºC. Chips were soaked in acetonitrile overnight to prevent solvent loss from droplets. Channels fabricated into PDMS devices were 100 µm in depth. Droplets were flowed in from a 100 µm wide channel that expanded to 200 µm wide at the point of intersection with the reagent addition channel, which was 100 µm wide. The final device was 200 µm wide at all openings to accommodate direct insertion with 360 µm o.d. tubing. Channels were wetted with PFD to help ease insertion of tubing (Supplementary Figure 8).

Reagent Addition Chip Screen

In-droplet photoreactions with online MS analysis were performed using reagent addition chips. Reagent addition PDMS chips were fabricated to enable trains of droplets to be imported from 100 µm i.d. PFA tubing, flowed through an addition region to receive reagents, and then be

exported to 150 µm i.d. PFA tubing for irradiation and ESI-MS analysis. Larger 150 µm i.d. tubing was used for the droplets post-addition as the larger volume droplets were sometimes unstable in the 100 µm i.d. tubing (*2*). Consistent addition of reagent to 4 nL acetonitrile droplets was achieved with the employed geometry (Supplementary Figure 8). By keeping droplet flow consistent (800 nL/min), the amount of reagent added to each droplet was controllable by the flow of the reagent stream. The reagent solution for the trifluoromethylation reaction, consisting of photocatalyst, pyridine *N*-oxide, and TFAA, (see General Procedure A) was utilized in the demonstration of the reagent addition device, as it showed a deep yellow color. At 100 nL/min reagent flow, the final droplets were composed of 33 ± 2% added reagent, while at 200 nL/min reagent flow created droplets with 45 ± 4% added reagent, showing consistent addition to droplets at both flow rates. Also tested for this geometry was the carry-over between droplets.

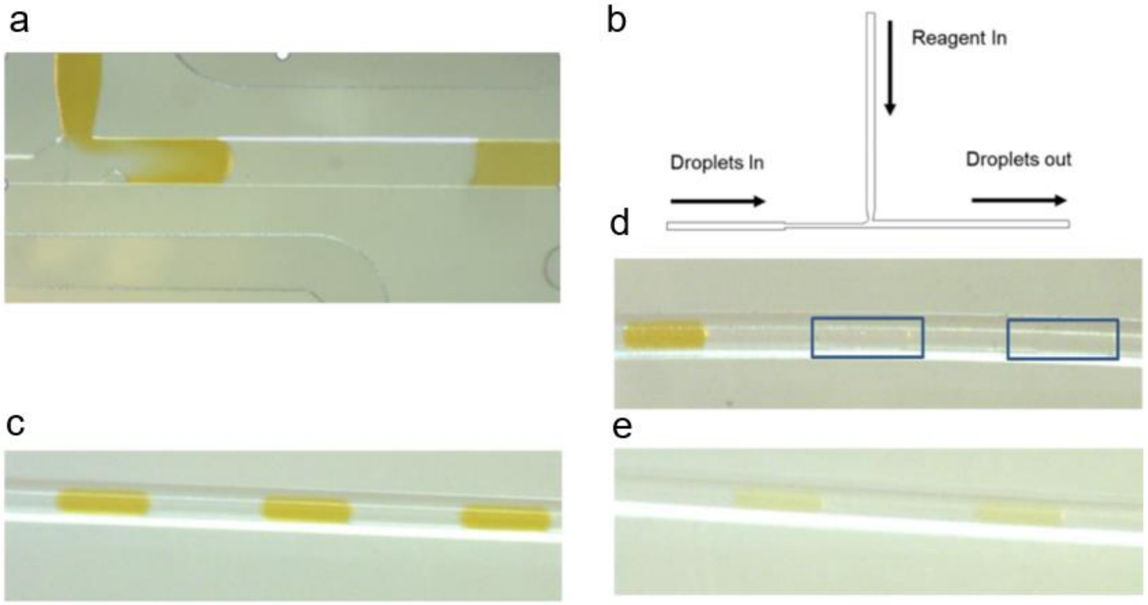

Supplementary Figure 8. Reagent addition device operation. Samples used contained either blank acetonitrile (clear, colorless) or trifluoromethylation reagent solution (dark yellow). Droplets were 4 nL initially, with 12 nL PFD spacing. (a) Device in operation. Each incoming droplet from the left received solution from the upper channel and moved right to export. Additional channels on top and bottom of channel were placed for optional saltwater electrodes. This feature was not necessary, as droplets coalesced with reagent stream without application of electric field. (b) Final design of reagent addition device, with electrodes removed. (c) Droplets post addition in PFA tubing. Droplet flow in was 800 nL/min, while reagent addition flow was 200 nL/min. Output droplets were found to contain 45 ± 4% added reagent. (d) Carry-over evaluation. Droplets were generated from either blank acetonitrile or reagent mixture. Blank acetonitrile was added to each droplet. Blank acetonitrile droplets (highlighted by blue boxes) flowed through addition device after reagent droplets show no coloration, indicating that very low carry-over exists during the operation of the reagent addition device. (e) Droplets at 6% reagent, showing significantly more yellow coloration than the blank droplets in (d).

To test for this, droplets were made in 10 x 10 units, alternating between being composed of either blank acetonitrile or trifluoromethylation reagent solution, and blank acetonitrile used as the addition stream. As the reagent addition stream is now colorless, any material carry-over from trifluoromethylation reagent droplets into the addition stream will lead to a yellow hue in the proceeding droplets. From this approach, blank acetonitrile droplets following trifluoromethylation reagent droplets had no observable yellow coloration, showing that this geometry can be performed with minimal carry-over between droplets (Supplementary Figure 8).

The above system was applied to the Smiles-Truce rearrangement described in the main manuscript. 4 nL acetonitrile droplets containing the 4-CN sulfonylacetamide substrate and trans-anethole (Supplementary Figure 9) segmented by 12 nL PFD were flowed through the reagent addition device at 800 nL/min, with 200 nL/min reagent addition flow, creating 7 nL full reaction droplets for irradiation and analysis. Irradiation time was approximately 7 min, calculated from the volume of the tubing contained within the reactor and the 1000 nL/min volumetric flow rate. Analysis of droplets post-irradiation at 20 droplets/min not only showed that product had formed, but that the formation was highly consistent across all the droplet samples (Supplementary Figure 9). To confirm that signal was a result of in-droplet chemistry, premixed reaction mixture was made into droplets and analyzed without irradiation. Minimal signal was observed, with the droplet samples barely distinguishable from background noise. These results indicate the successful application of our system for performing and analyzing in-droplet photoredox reactions.

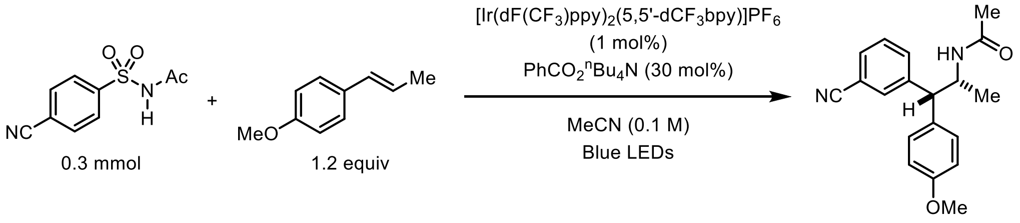

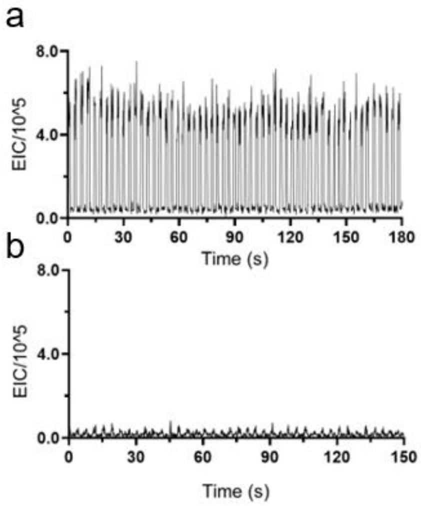

Supplementary Figure 9. In-droplet Smiles-Truce rearrangement reaction using reagent addition device. MS traces represent the m/z of the Smiles-Truce rearrangement product (m/z = 309). (a) Droplet samples processed with online flow reactor. (b) Control samples of (a) which was not exposed to blue LED irradiation. Minimal signal was observed, indicating that results from (a) are the result of in-droplet chemistry.

Benchmarking Analysis of Reaction Screening Platforms

The following table shows a comparison of our droplet microfluidic HTE platform with other screening platforms in the literature. Figures of merit are based on references published and highlight the differences of the current platform from previous work.

Screening System Reference	Reaction Vessel Volume (µL)	Analytical Throughput (acquisition rate in samples/s)	Material Usage (nmol per reaction)	Flow vs. Plate-based	Analytical Technique	Reaction Type
This work	0.005	0.3 [a]	0.5	Flow*	ESI-MS	Photochemical
6	5	0.02	100-300	Flow*	UPLC-MS	Suzuki Cross Coupling
7	15	n/a	200-800	Flow	HPLC-MS	Photochemical
8	8	2	0.02	Flow	ESI-MS	Diels-Alder reaction (limited to aqueous solvents)
9	0.050	0.3-1.67	1-5	Plate-based	DESI-MS	Suzuki Cross Coupling
10	1-10	0.05-0.2	50	Plate-based	MISER HPLC-MS	Suzuki Cross Coupling
11	1-10	0.008-0.017	50	Plate-based	UPLC-MS	Suzuki Cross Coupling

***Directly compatible with plate-based compound libraries**

Supplementary Table 1. Comparison of our droplet microfluidics screening system against several representative plate-based and flow-based screening systems in the literature. [a] Overall throughput for this system is approximately 0.03 samples/s (350 samples/200 min), given a sample prep time of 120 min/350 samples, residence time of 60 min/350 samples, and analytical sample time of ~20 min.

<u>Compound Characterization</u>

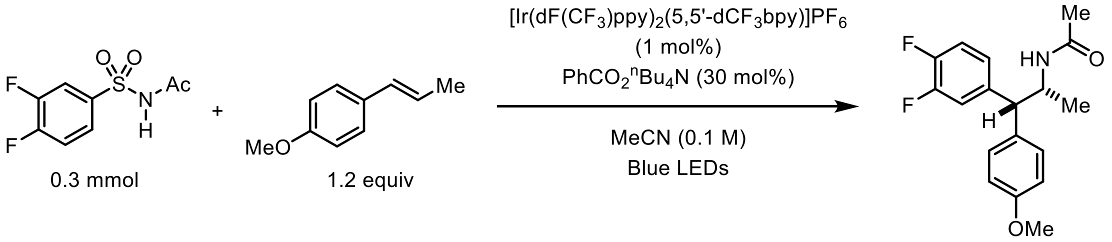

N-(1-(3,4-difluorophenyl)-1-(4-methoxyphenyl)propan-2-yl)acetamide (5)

The reaction was run according to General Procedures B and E on 0.1 mmol scale. The crude reaction was purified by column chromatography (20% to 70% ethyl acetate in hexanes; 1% spiked acetic acid in ethyl acetate) to afford the title compound (10 mg, 31%) as a light yellow foam. Rf = 0.20 (ethyl acetate/hexanes 7:3 with 1% acetic acid in ethyl acetate; UV). ^{1}H NMR (700 MHz, $CDCl_3$) δ 7.16 – 7.10 (m, 2H), 7.06 (qd, *J* = 9.4, 3.5 Hz, 2H), 7.02 (s, 1H), 6.87 – 6.78 (m, 2H), 5.19 (s, 1H), 4.82 – 4.74 (m, 1H), 3.77 (d, *J* = 3.1 Hz, 3H), 3.75 (d, *J* = 9.3 Hz, 1H), 1.86 – 1.81 (m, 3H), 1.13 – 1.06 (m, 3H). ^{13}C NMR (176 MHz, $CDCl_3$) δ 169.3, 158.5, 150.8 (d, *J* = 12.7 Hz), 149.7 (d, *J* = 12.3 Hz), 149.4 (d, *J* = 13.2 Hz), 148.3 (d, *J* = 12.6 Hz), 139.4, 133.3, 129.1, 123.6, 117.3 (d, *J* = 17.0 Hz), 116.9 (d, *J* = 17.4 Hz), 114.2, 56.5, 55.2, 47.7, 23.3, 20.1. HRMS (ESI) m/z $[M + H]^+$ calcd for: $C_{18}H_{19}F_2NO_2$: 320.1457; found: 320.1455. IR (neat): v = 3276, 3066, 2932, 2842, 1643, 1609, 1553, 1510, 1455, 1432, 1372, 1282, 1251.

^{1}H NMR (700 MHz, $CDCl_3$) for 5

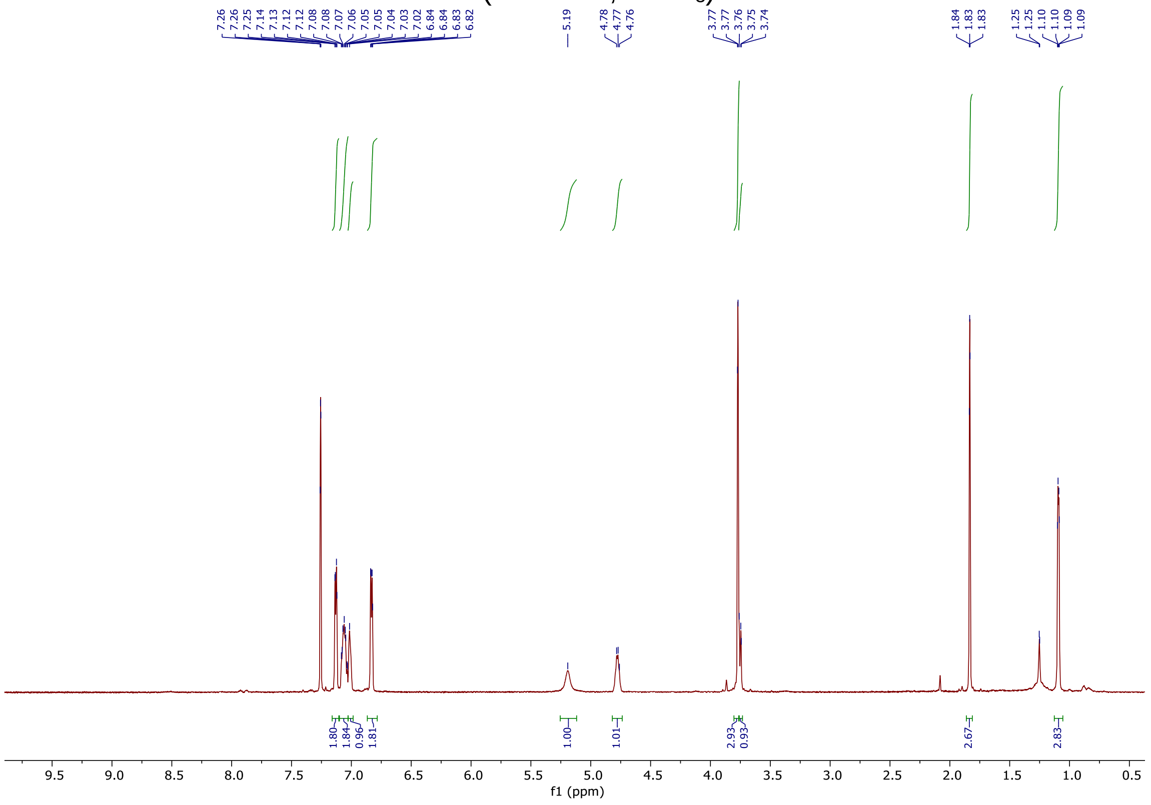

^{13}C NMR (176 MHz, $CDCl_3$) for 5

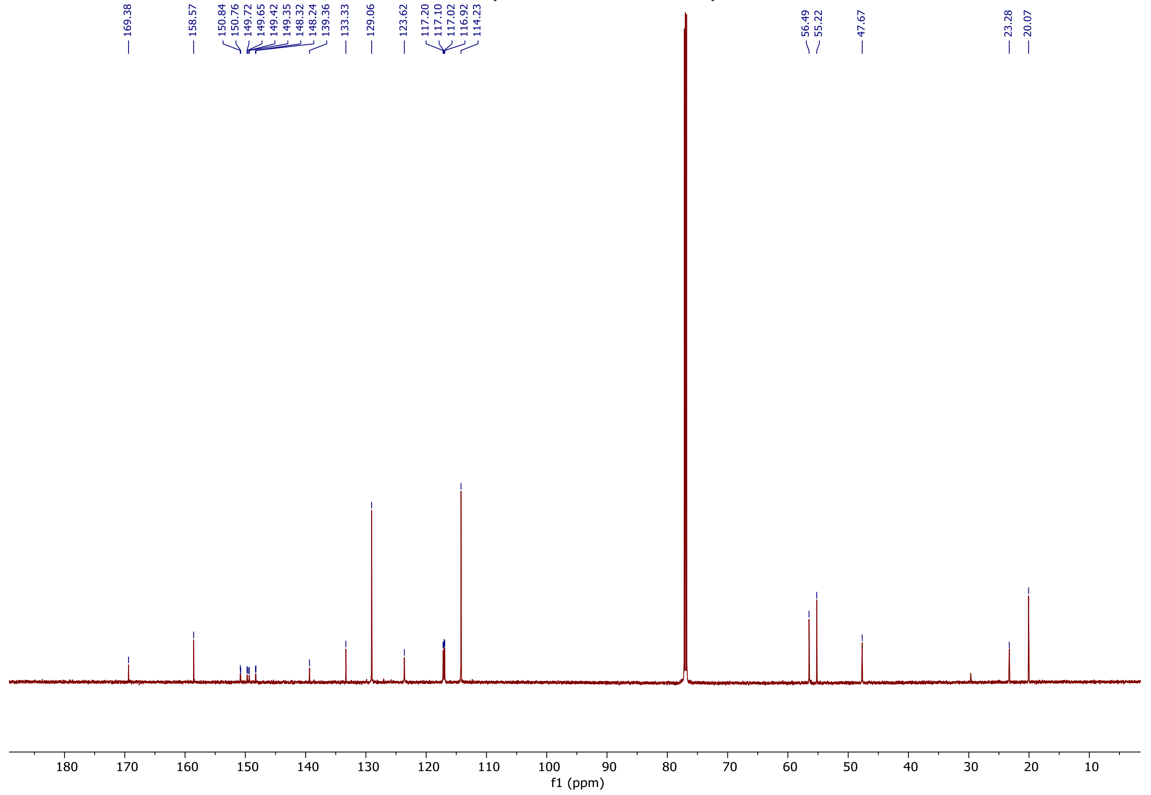

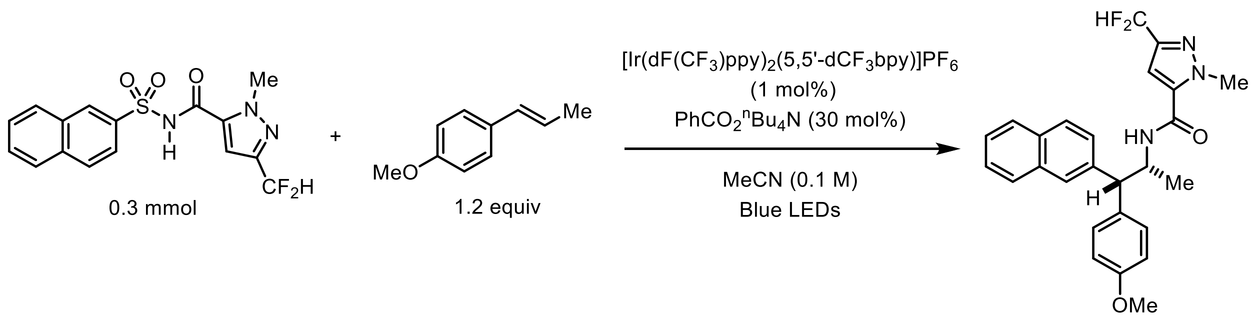

3-(difluoromethyl)-*N*-1-(4-methoxyphenyl)-1-(naphthalen-2-yl)propan-2-yl)-1-methyl-1H-pyrazole-5-carboxamide (6)

The reaction was run according to General Procedures B and E on 0.1 mmol scale. The crude reaction was purified by column chromatography (20% to 70% ethyl acetate in hexanes; 1% spiked acetic acid in ethyl acetate) to afford the title compound (16 mg, 35%) as a light yellow foam. Rf = 0.24 (ethyl acetate/hexanes 7:3 with 1% acetic acid in ethyl acetate; UV). ^{1}H NMR (500 MHz, $CDCl_3$) δ 8.17 (d, *J* = 8.6 Hz, 1H), 7.80 (d, *J* = 8.1 Hz, 1H), 7.73 – 7.66 (m, 2H), 7.59 (s, 1H), 7.51 – 7.39 (m, 3H), 7.30 (d, *J* = 8.3 Hz, 2H), 6.80 (dd, *J* = 6.3, 3.9 Hz, 2H), 6.51 (t, *J* = 54.2 Hz, 2H), 6.03 (s, 1H), 5.12 – 5.02 (m, 1H), 4.72 (d, *J* = 10.2 Hz, 1H), 3.81 (s, 3H), 3.73 (d, *J* = 1.2 Hz, 3H), 1.27 (d, *J* = 6.5 Hz, 3H). ^{13}C NMR (176 MHz, $CDCl_3$) δ 160.5, 158.3, 142.8 (t, *J* = 27.6 Hz), 137.4, 134.1, 134.1, 133.7, 132.0, 129.5, 128.9, 127.2, 126.0, 125.5, 125.2, 124.3, 123.2, 116.9, 114.0, 111.0 (t, *J* = 233.9 Hz), 55.2, 51.7, 48.5, 39.3, 20.5. ^{19}F NMR (471 MHz, $CDCl_3$) δ -110.14 (dd, *J* = 1476.3, 54.1 Hz), -110.14 (dd, *J* = 862.4, 54.5 Hz). HRMS (ESI) m/z $[M + H]^+$ calcd for: $C_{26}H_{25}F_2N_3O_2$: 450.1988; found: 450.1991. IR (neat): ν = 3313, 2981, 1633, 1609, 1556, 1510, 1453, 1398, 1355, 1302, 1252, 1177.

^{1}H NMR (500 MHz, $CDCl_3$) for 6

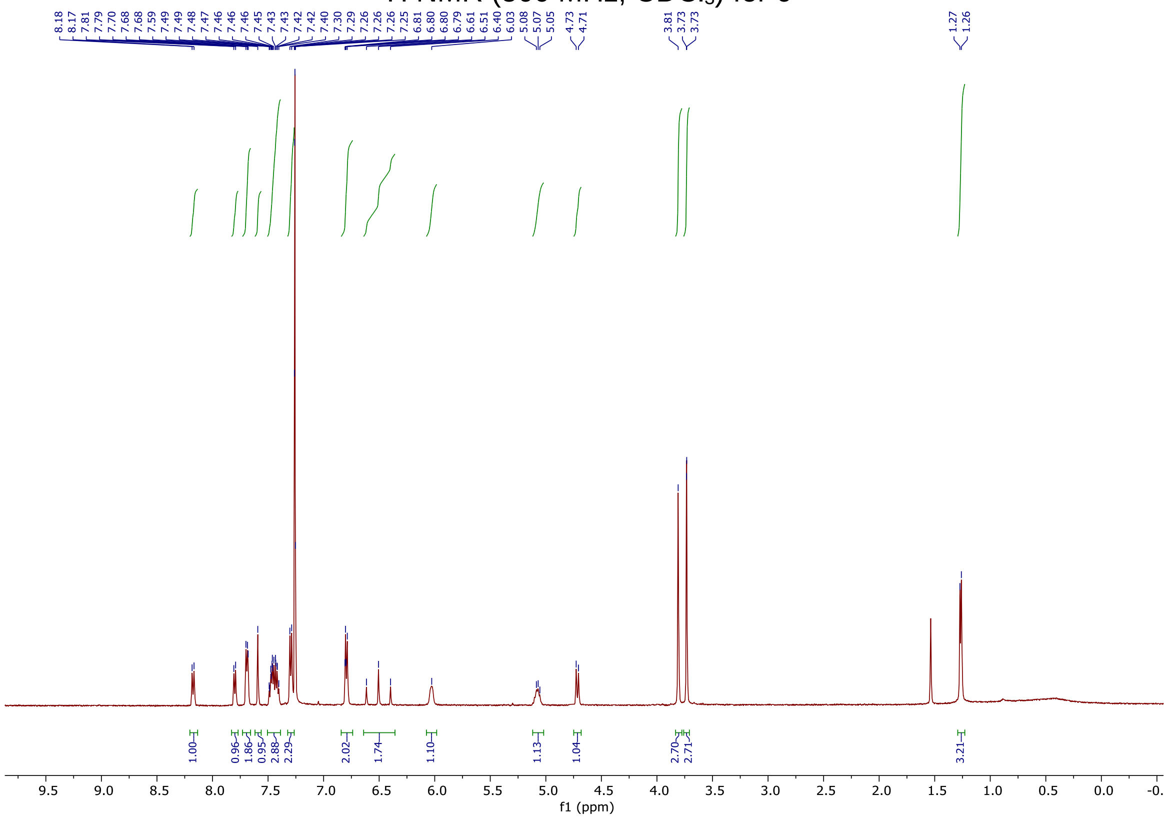

^{13}C NMR (176 MHz, $CDCl_3$) for 6

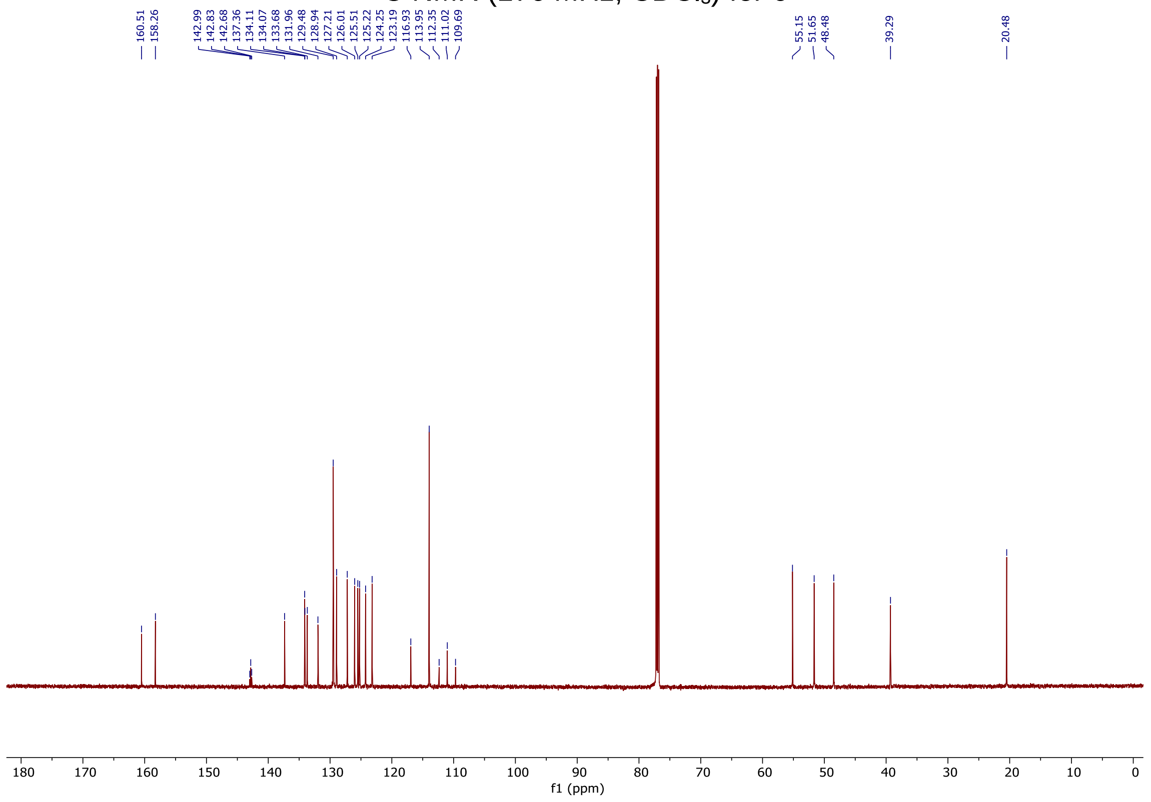

^{19}F NMR (471 MHz, $CDCl_3$) for 6

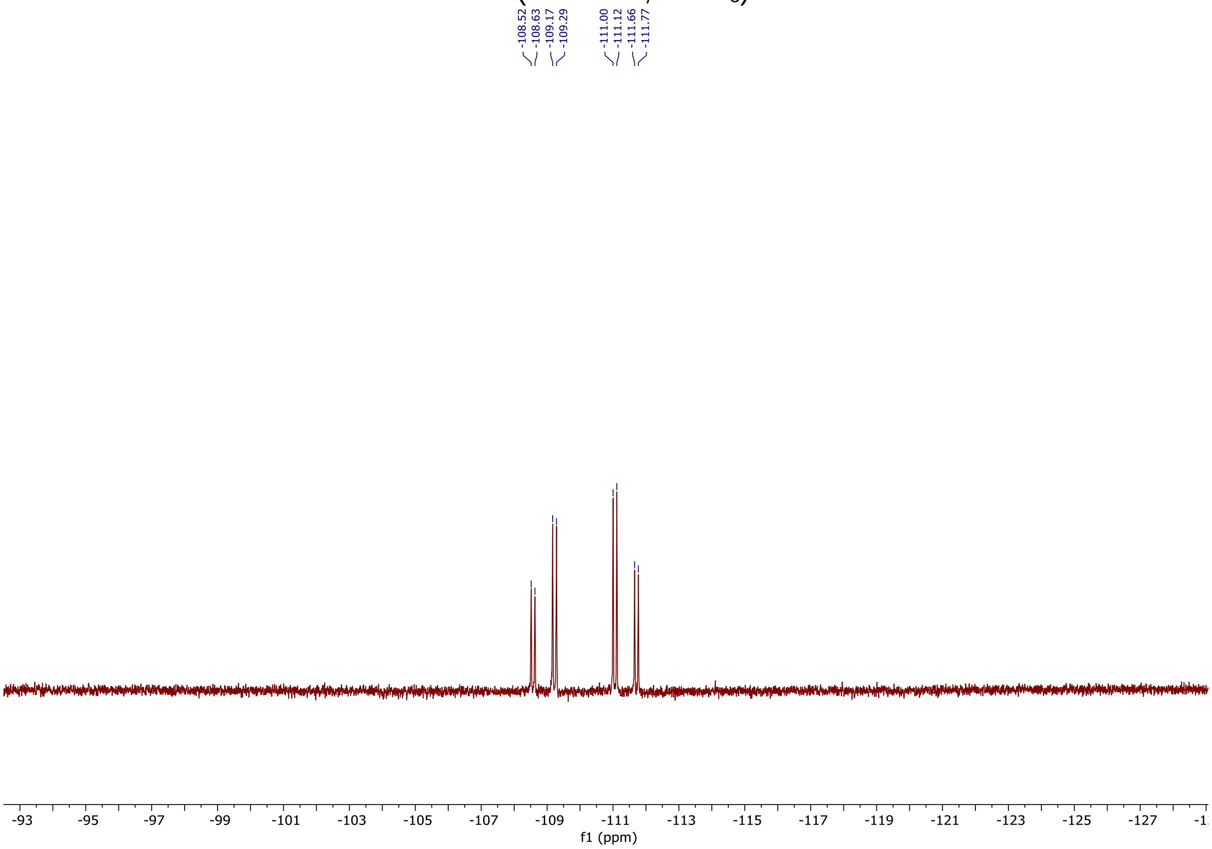

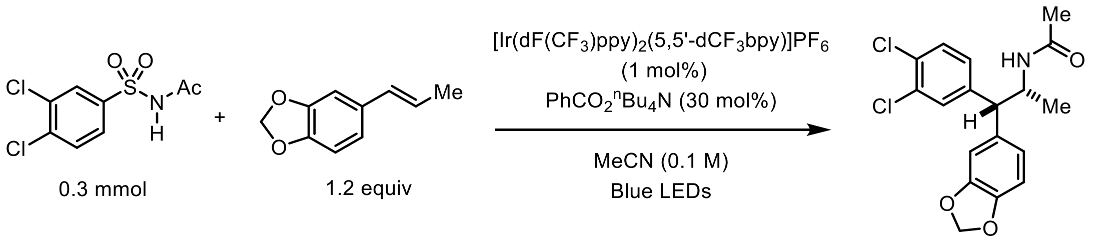

N-1-(benzo[d][1,3]dioxol-5-yl)-1-(3,4-dichlorophenyl)propan-2-yl)acetamide (7)

The reaction was run according to General Procedures B and E on 0.1 mmol scale. The crude reaction was purified by column chromatography (20% to 70% ethyl acetate in hexanes; 1% spiked acetic acid in ethyl acetate) to afford the title compound (10 mg, 27%) as a light yellow foam. Rf = 0.25 (ethyl acetate/hexanes 7:3 with 1% acetic acid in ethyl acetate; UV). ^{1}H NMR (500 MHz, $CDCl_3$) δ 7.37 – 7.31 (m, 2H), 7.16 (dd, *J* = 8.3, 2.2 Hz, 1H), 6.73 (d, *J* = 7.8 Hz, 1H), 6.71 – 6.66 (m, 2H), 5.95 – 5.89 (m, 2H), 5.13 (d, *J* = 9.2 Hz, 1H), 4.76 (d, *J* = 6.4 Hz, 1H), 3.70 (d, *J* = 10.3 Hz, 1H), 1.83 (s, 3H), 1.11 (d, *J* = 6.6 Hz, 3H). ^{13}C NMR (176 MHz, $CDCl_3$) δ 169.2, 148.1, 146.7, 142.3, 134.9, 132.4, 130.6, 130.5, 130.2, 126.9, 121.3, 108.5, 108.2, 101.1, 57.1, 47.3, 23.4, 20.2. HRMS (ESI) m/z $[M + H]^+$ calcd for: $C_{18}H_{17}Cl_2NO_3$: 366.0658; found: 366.0655. IR (neat): ν = 3278, 3077, 2927, 1726, 1649, 1548, 1503, 1489, 1443, 1373, 1330, 1245, 1230.

^1H NMR (500 MHz, $CDCl_3$) for 7

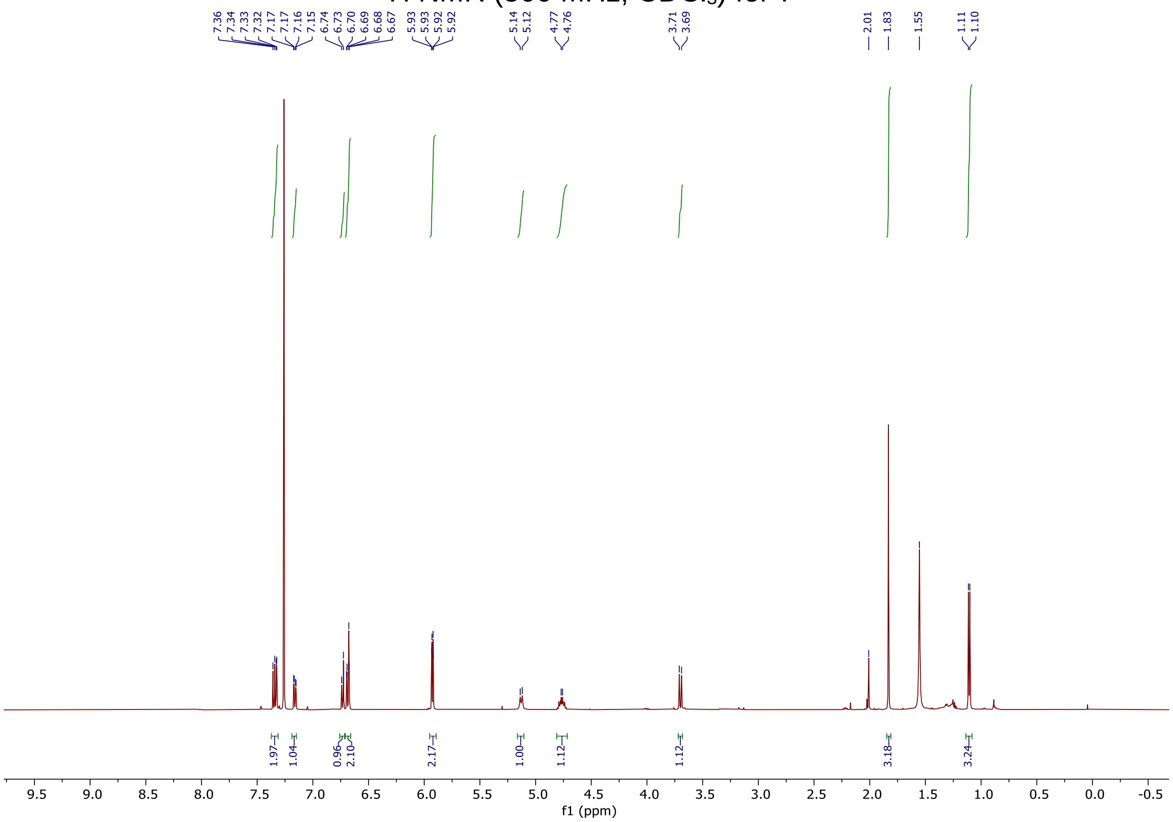

^{13}C NMR (176 MHz, $CDCl_3$) for 7

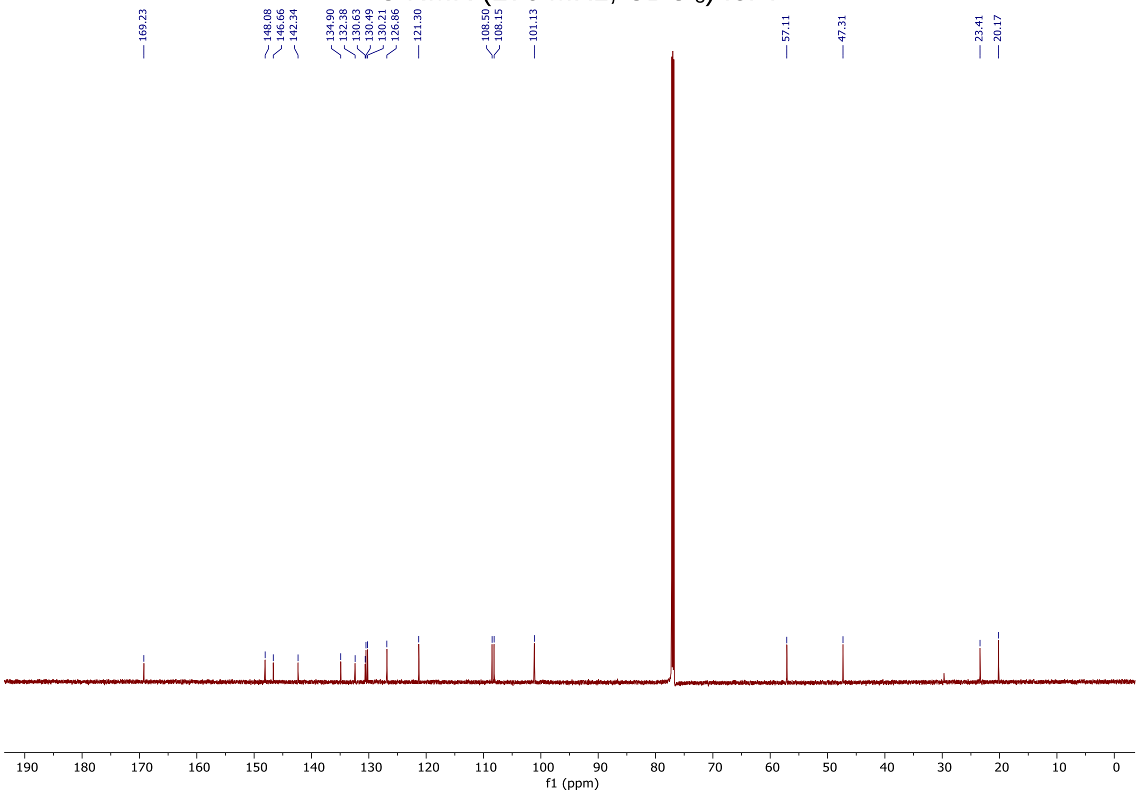

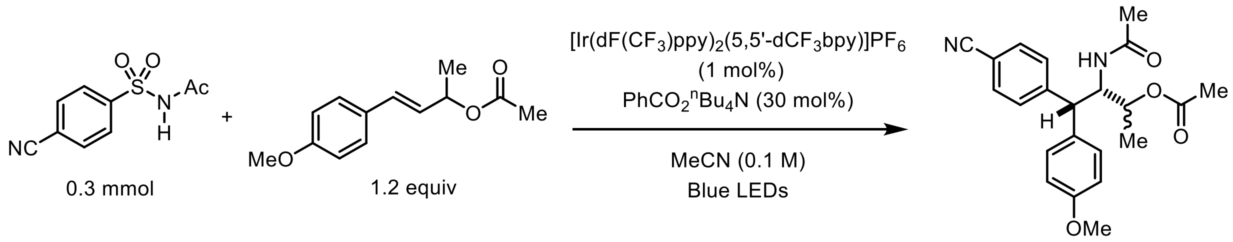

3-acetamido-4-(4-cyanophenyl)-4-(4-methoxyphenyl)butan-2-yl acetate (8)

The reaction was run according to General Procedures B and E on 0.1 mmol scale. The crude reaction was purified by column chromatography (20% to 70% ethyl acetate in hexanes; 1% spiked acetic acid in ethyl acetate) to afford the title compound (5 mg, 13%) as a light yellow foam. Rf = 0.22 (ethyl acetate/hexanes 7:3 with 1% acetic acid in ethyl acetate; UV). A 1:1.5 ratio of diastereomers (minor diastereomer 8A and major diastereomer 8B) was observed by ^{1}H and ^{13}C NMR. ^{1}H NMR (700 MHz, $CDCl_3$) δ 7.57 – 7.54 (m, 2H, 8A), 7.55 – 7.52 (m, 2H, 8B), 7.43 (d, *J* = 7.8 Hz, 2H, 8A), 7.40 – 7.35 (m, 2H, 8B), 7.25 – 7.22 (m, 2H, 8B), 7.07 (d, *J* = 8.1 Hz, 2H, 8A), 6.87 – 6.83 (m, 2H, 8B), 6.83 – 6.80 (m, 2H, 8A), 5.43 (d, *J* = 10.3 Hz, 1H, 8A), 5.29 (d, *J* = 5.0 Hz, 1H, 8B), 5.27 (s, 1H, 8A), 5.14 (d, *J* = 10.3 Hz, 1H, 8B), 4.90 (t, *J* = 11.0 Hz, 1H, 8A), 4.83 (d, *J* = 5.3 Hz, 1H, 8B), 4.74 (d, *J* = 6.6 Hz, 1H, 8A), 3.94 (d, *J* = 11.8 Hz, 1H, 8A), 3.85 (d, *J* = 11.2 Hz, 1H, 8B), 3.76 (d, *J* = 1.7 Hz, 3H, 8B), 3.75 (d, *J* = 1.8 Hz, 3H, 8A), 2.14 (d, *J* = 1.8 Hz, 3H, 8A), 1.96 (d, *J* = 1.8 Hz, 3H, 8B), 1.85 (d, *J* = 1.8 Hz, 3H, 8A), 1.81 (d, *J* = 1.8 Hz, 3H, 8B), 1.18 – 1.16 (m, 3H, 8A), 1.16 – 1.13 (m, 3H, 8B). ^{13}C NMR (176 MHz, $CDCl_3$) δ 170.6, 170.0, 169.8, 169.4, 158.9, 158.9, 147.5, 147.4, 132.4, 132.3, 131.9, 131.6, 128.8, 128.7, 128.7, 128.5, 118.8, 118.7, 114.7, 114.6, 110.5, 110.4, 70.2, 70.1, 55.2, 55.2, 54.3, 53.9, 53.3, 52.1, 23.2, 23.1, 21.2, 21.1, 17.4, 13.8. HRMS (ESI) m/z $[M + H]^+$ calcd for $C_{22}H_{24}N_2O_4$: 381.1809; found: 381.1806. IR (neat): v = 3297, 2982, 2228, 1731, 1656, 1609, 1582, 1539, 1512, 1463, 1443, 1371, 1302, 1241.

^{1}H NMR (700 MHz, $CDCl_3$) for 8A and 8B

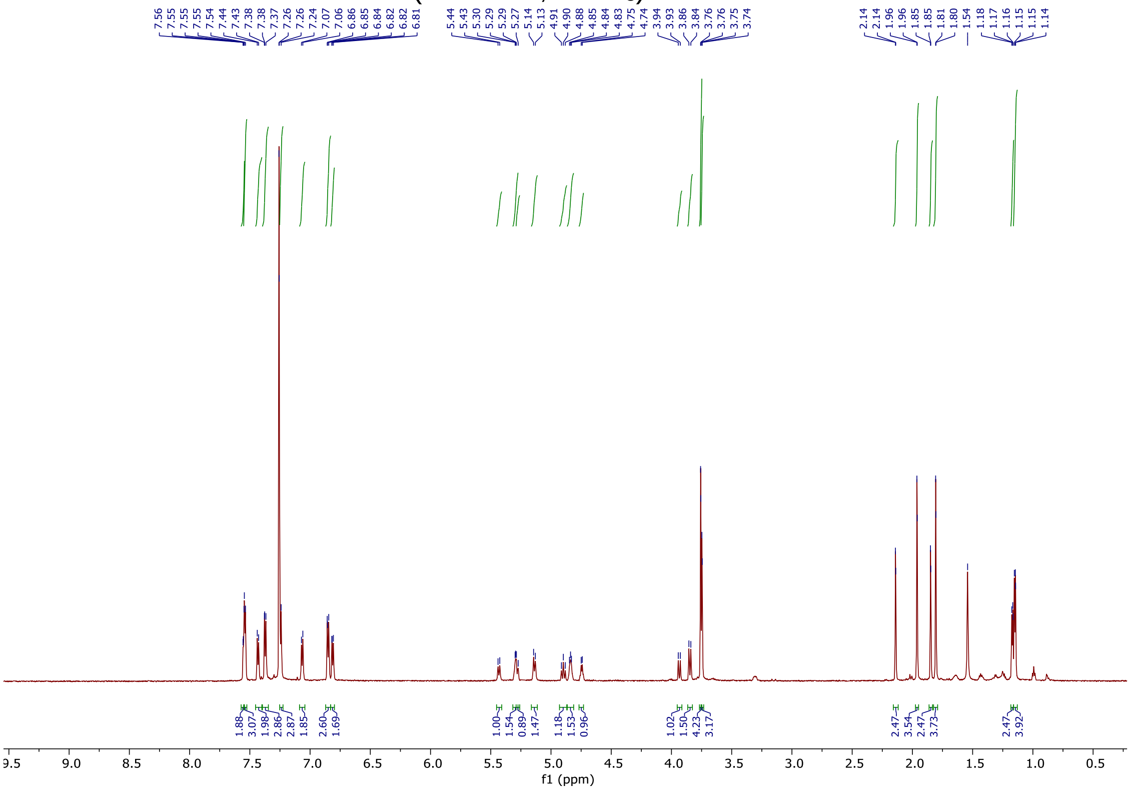

^{13}C NMR (176 MHz, $CDCl_3$) for 8A and 8B

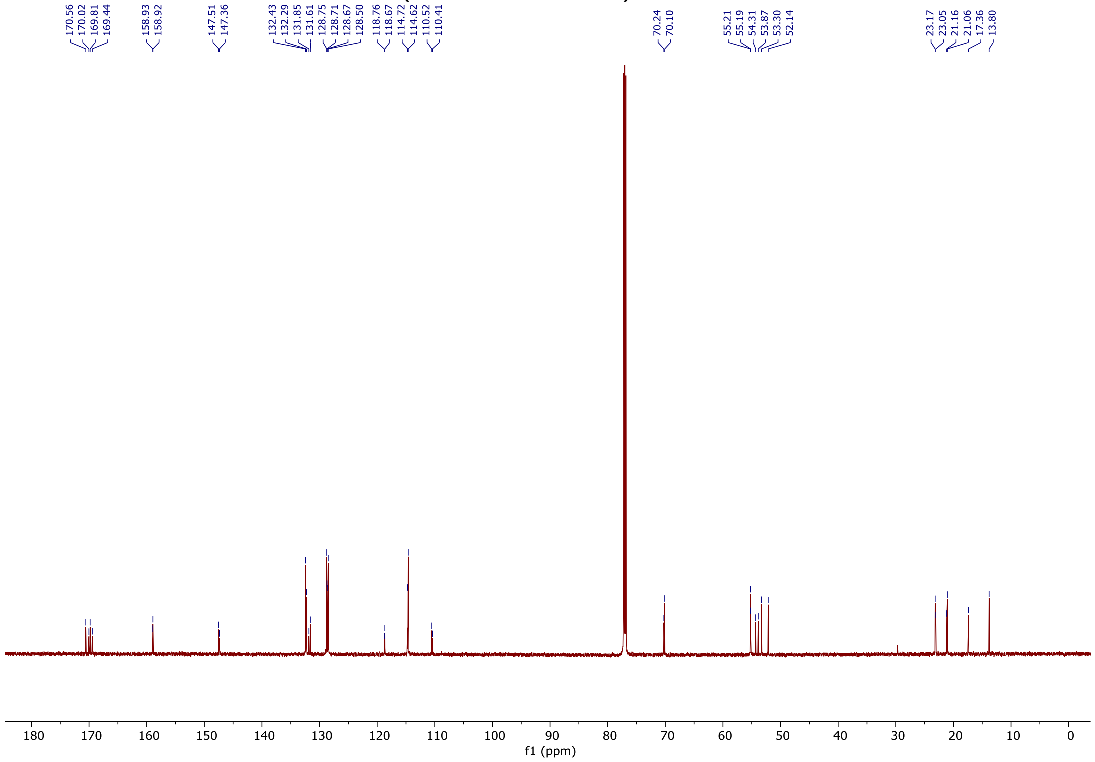

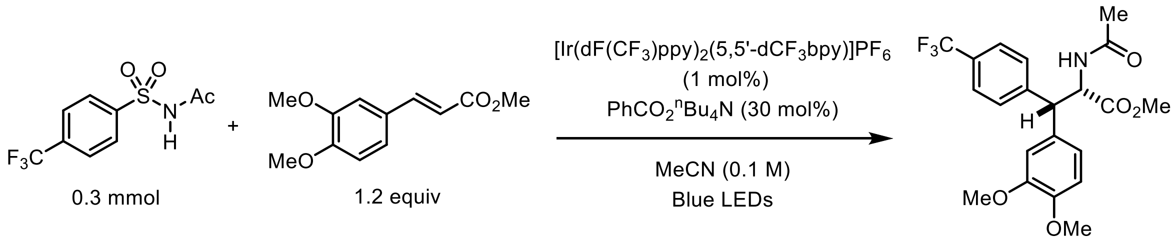

Methyl-(2-acetamido-3-(3,4-dimethoxyphenyl)-3-(4-(trifluoromethyl)phenyl)propanoate (9)

The reaction was run according to General Procedures B and E on 0.1 mmol scale. The crude reaction was purified by column chromatography (20% to 70% ethyl acetate in hexanes; 1% spiked acetic acid in ethyl acetate) to afford the title compound (7 mg, 15%) as a light yellow foam. Rf = 0.23 (ethyl acetate/hexanes 7:3 with 1% acetic acid in ethyl acetate; UV). ^{1}H NMR (700 MHz, $CDCl_3$) δ 7.57 (d, *J* = 8.0 Hz, 2H), 7.38 (d, *J* = 7.9 Hz, 2H), 6.80 – 6.74 (m, 2H), 6.72 (s, 1H), 5.67 (d, *J* = 9.2 Hz, 1H), 5.45 (t, *J* = 9.2 Hz, 1H), 4.40 (d, *J* = 9.1 Hz, 1H), 3.84 (s, 3H), 3.82 (s, 3H), 3.53 (d, *J* = 1.8 Hz, 3H), 1.90 (d, *J* = 1.9 Hz, 3H). ^{13}C NMR (176 MHz, $CDCl_3$) δ 172.2, 169.8, 149.0, 148.4, 143.9, 131.4, 129.5 (q, *J* = 32.6 Hz), 128.6, 125.7 (q, *J* = 3.7 Hz), 124.0 (q, *J* = 272.0 Hz), 120.5, 111.3, 111.2, 55.9, 55.8, 55.0, 53.2, 52.3, 23.1. ^{19}F NMR (471 MHz, $CDCl_3$) δ -62.59. HRMS (ESI) m/z $[M + H]^+$ calcd for $C_{21}H_{22}F_3NO_5$: 426.1523; found: 426.1521. IR (neat): v = 3282, 2981, 1744, 1659, 1513, 1464, 1326, 1250, 1122.

^{1}H NMR (700 MHz, $CDCl_3$) for 9

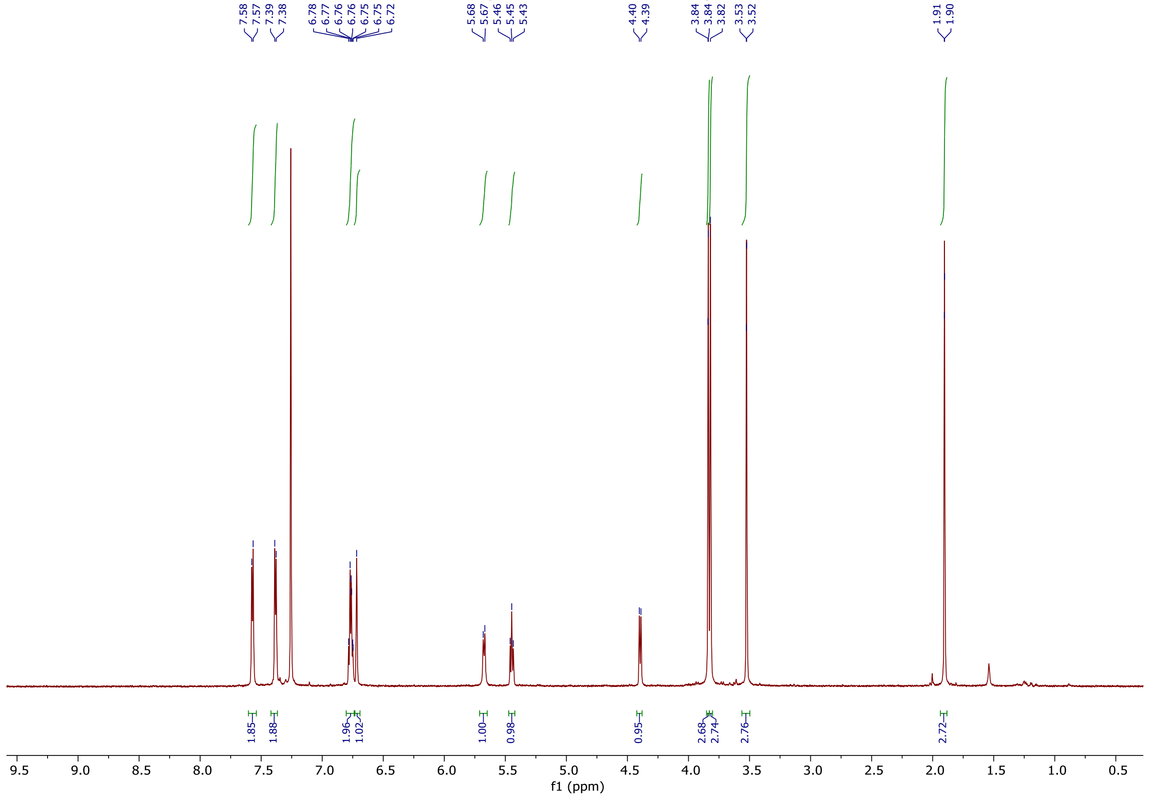

^{13}C NMR (176 MHz, $CDCl_3$) for 9

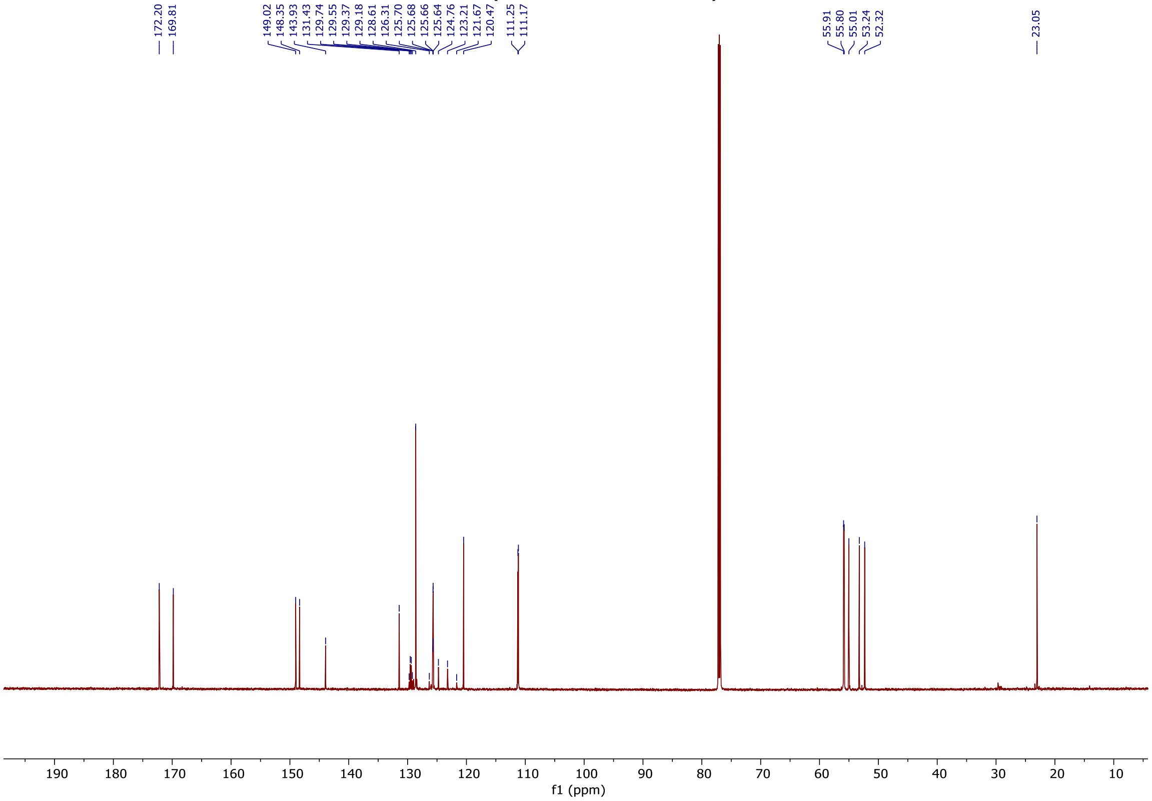

^{19}F NMR (471 MHz, $CDCl_3$) for 9

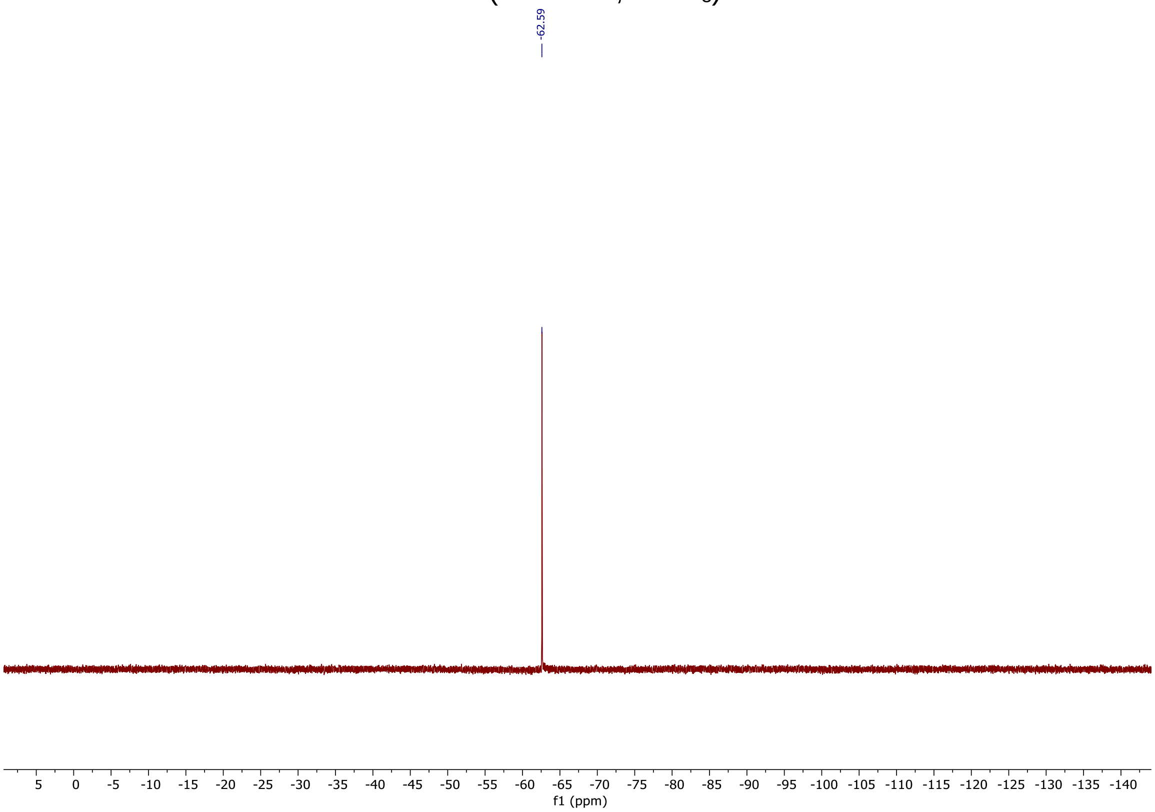

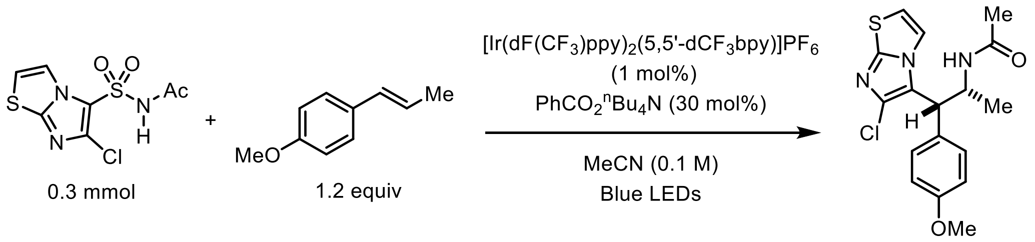

N-1-(6-chloroimidazo[2,1-b]thiazol-5-yl)-1-(4-methoxyphenyl)propan-2-yl)acetamide (10)

The reaction was run according to General Procedures B and E on 0.1 mmol scale. The crude reaction was purified by column chromatography (20% to 70% ethyl acetate in hexanes; 1% spiked acetic acid in ethyl acetate) to afford the title compound (25 mg, 68%) as a light yellow foam. Rf = 0.19 (ethyl acetate/hexanes 7:3 with 1% acetic acid in ethyl acetate; UV). ^{1}H NMR (700 MHz, $CDCl_3$) δ 7.24 (d, *J* = 6.6 Hz, 2H), 7.17 (d, *J* = 4.4 Hz, 1H), 6.86 (d, *J* = 8.3 Hz, 2H), 6.74 (d, *J* = 4.5 Hz, 1H), 5.33 (d, *J* = 9.2 Hz, 1H), 5.00 (q, *J* = 8.4 Hz, 1H), 4.34 (d, *J* = 9.4 Hz, 1H), 3.78 (d, *J* = 2.6 Hz, 3H), 1.80 (d, *J* = 2.7 Hz, 3H), 1.29 (d, *J* = 6.5 Hz, 3H). ^{13}C NMR (176 MHz, $CDCl_3$) δ 169.5, 158.8, 147.0, 131.9, 129.8, 129.0, 121.8, 118.5, 114.4, 112.1, 55.2, 46.8, 46.2, 23.4, 20.6. HRMS (ESI) m/z $[M + H]^+$ calcd for $C_{17}H_{18}ClN_3O_2S$: 364.0881; found: 364.0876. IR (neat): ν = 3269, 3081, 2880, 1655, 1611, 1552, 1512, 1458, 1372, 1293, 1250.

^{1}H NMR (700 MHz, $CDCl_3$) for 10

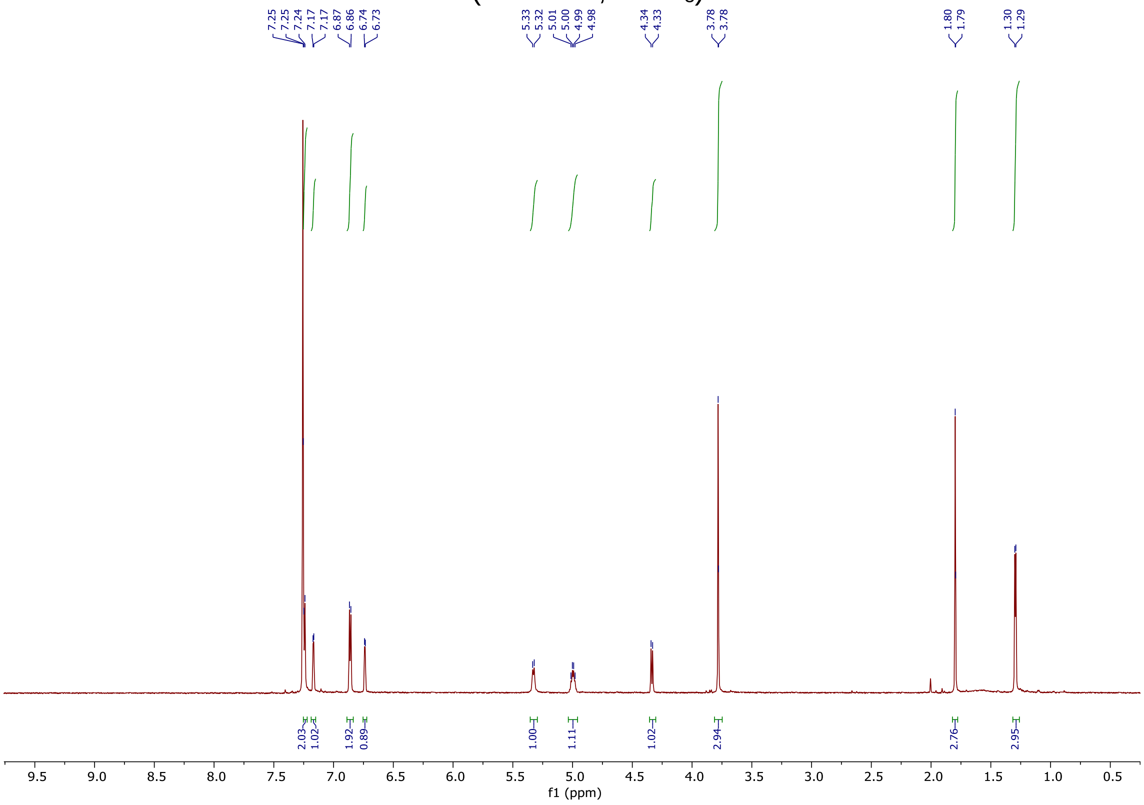

^{13}C NMR (176 MHz, $CDCl_3$) for 10

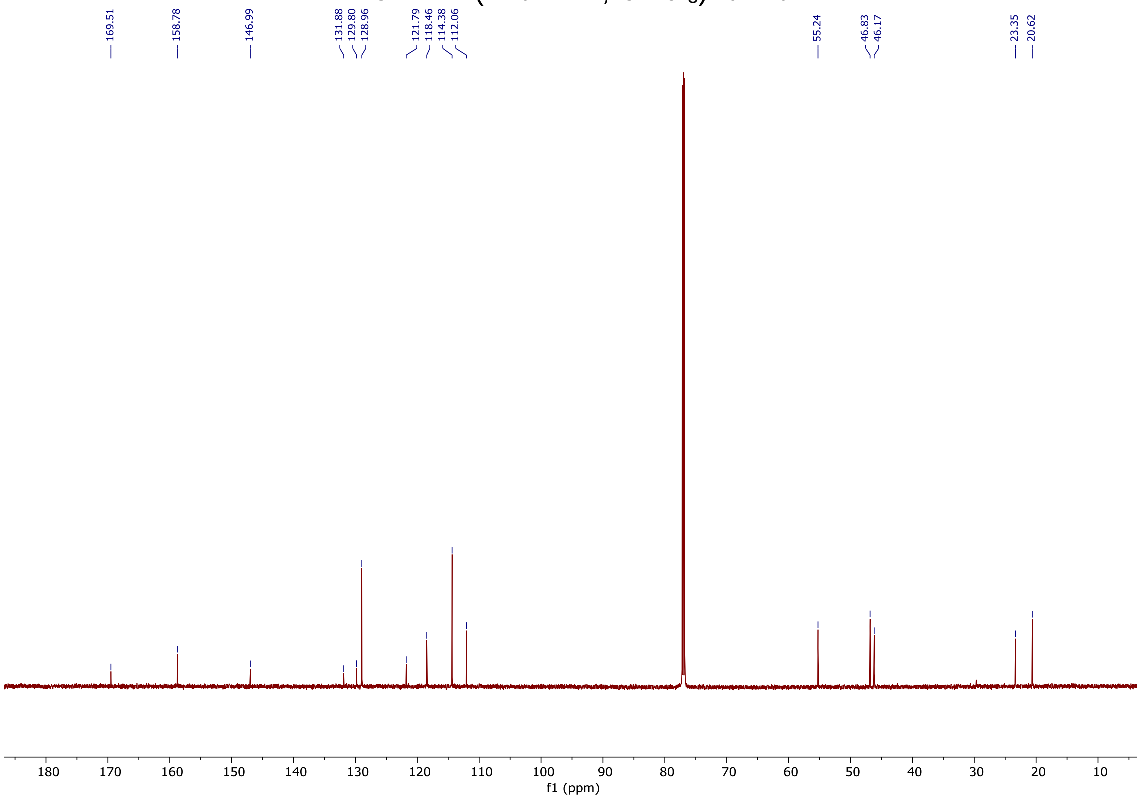

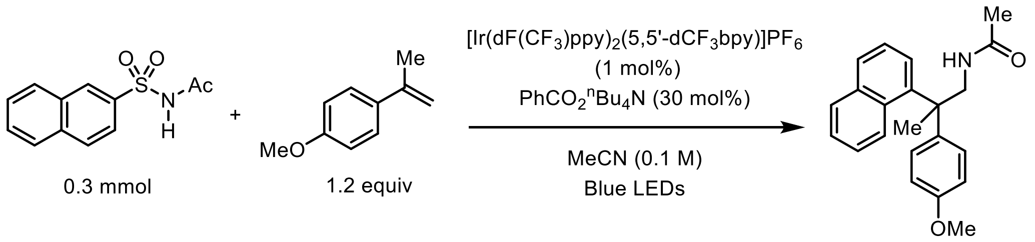

N-(2-(4-methoxyphenyl)-2-(naphthalen-1-yl)propyl)acetamide (11)

The reaction was run according to General Procedures B and E on 0.1 mmol scale. The crude reaction was purified by column chromatography (20% to 70% ethyl acetate in hexanes; 1% spiked acetic acid in ethyl acetate) to afford the title compound (7 mg, 20%) as a light yellow foam. Rf = 0.26 (ethyl acetate/hexanes 7:3 with 1% acetic acid in ethyl acetate; UV). ^{1}H NMR (700 MHz, $CDCl_3$) δ 7.82 (t, *J* = 9.6 Hz, 2H), 7.64 (d, *J* = 7.5 Hz, 1H), 7.50 (t, *J* = 7.8 Hz, 1H), 7.47 (d, *J* = 8.8 Hz, 1H), 7.34 (t, *J* = 7.3 Hz, 1H), 7.14 (t, *J* = 7.9 Hz, 1H), 7.10 (d, *J* = 8.4 Hz, 2H), 6.84 – 6.78 (m, 2H), 4.90 (s, 1H), 4.28 – 4.21 (m, 1H), 4.01 (dd, *J* = 13.3, 4.1 Hz, 1H), 3.78 (d, *J* = 2.5 Hz, 3H), 1.85 (d, *J* = 2.5 Hz, 3H), 1.78 (s, 3H). ^{13}C NMR (176 MHz, $CDCl_3$) δ 170.1, 157.9, 141.0, 139.5, 135.1, 130.9, 129.2, 128.5, 127.3, 127.0, 125.0, 125.0, 125.0, 124.9, 114.1, 55.2, 49.0, 47.0, 27.7, 23.4. $C_{22}H_{23}NO_2$: 334.1802; found: 334.1801. IR (neat): ν = 3317, 3050, 2931, 1656, 1609, 1609, 1509, 1463, 1440, 1397, 1371, 1289, 1249.

^{1}H NMR (700 MHz, $CDCl_3$) for 11

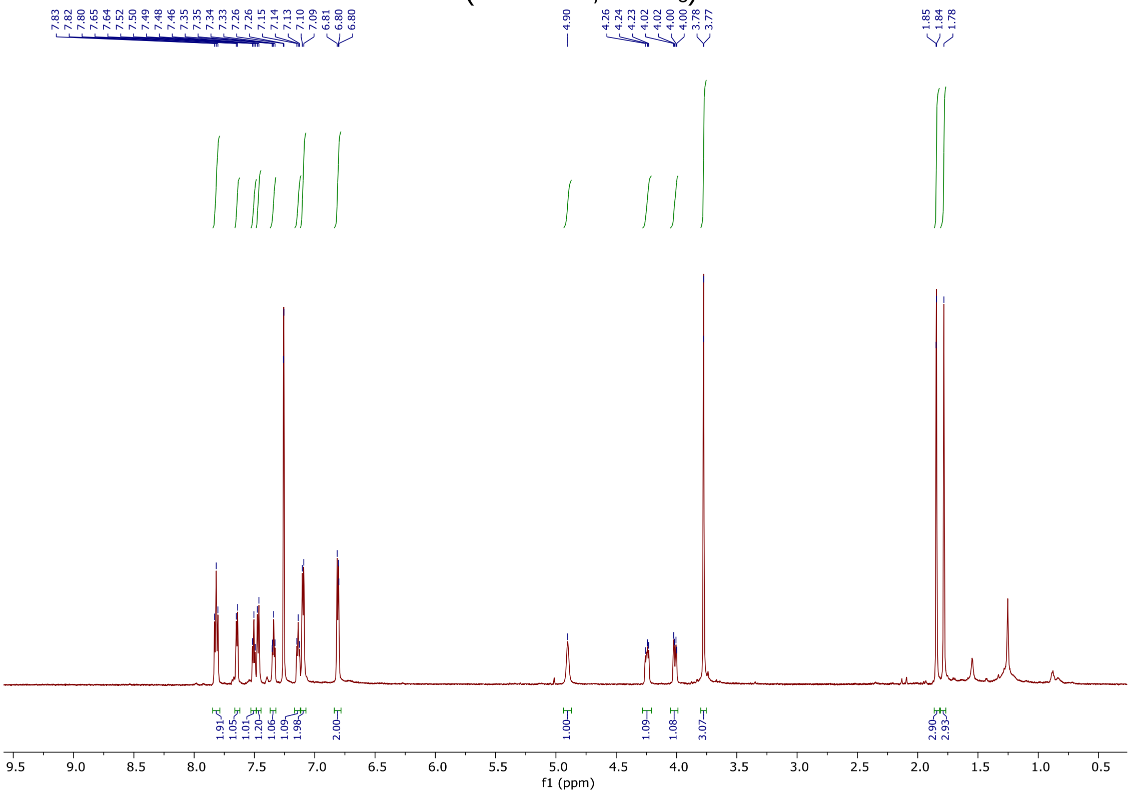

^{13}C NMR (176 MHz, $CDCl_3$) for 11

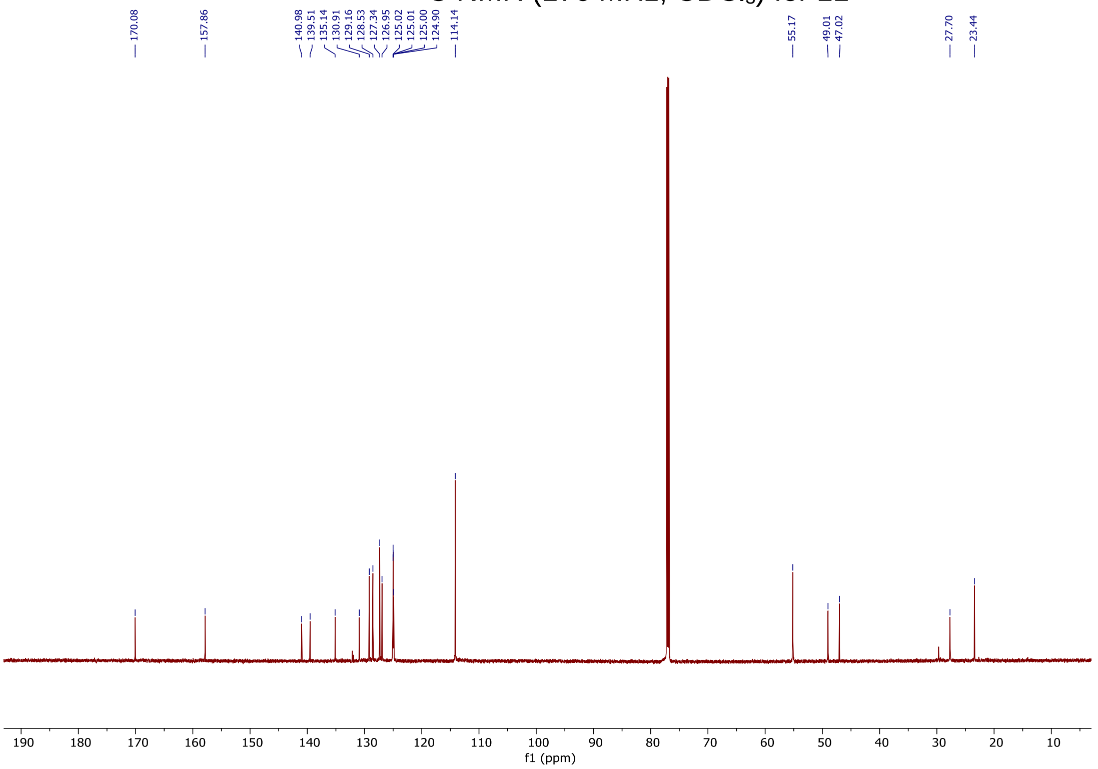

References

(1) Sun, S., Slaney, T. R. & Kennedy, R. T. Label-free screening of enzyme inhibitors at femtomole scale using segmented flow electrospray ionization mass spectrometry. *Anal. Chem.* **84**, 5794–5800 (2012).

(2) Chabert, M. *et al.*, Automated microdroplet platform for sample manipulation and polymerase chain reaction. *Anal. Chem.* **78**, 7722–7728 (2006).

(3) Pei, J. *et al.*, Analysis of samples stored as individual plugs in a capillary by electrospray ionization mass spectrometry. *Anal. Chem.* **81**, 6558–6561 (2009).

(4) Dugan, C. E. *et al.*, Multiplexed microfluidic enzyme assays for simultaneous detection of lipolysis products from adipocytes. *Anal. Bioanal. Chem.* **406**, 4851–4859 (2014).

(5) Hatakeyama, T., Chen, D. L. & Ismagilov, R. F. Microgram-scale testing of reaction conditions in solution using nanoliter plugs in microfluidics with detection by MALDI-MS. *J. Am. Chem. Soc.* **128**, 2518–2519 (2006).

(6) Perera, D. *et al*., A platform for automated nanomole-scale screening and micromole-scale synthesis in flow. *Science* **359**, 429–434 (2018).

(7) Coley, C. W., Abolhasani, M., Lin, H. & Jensen, K. F. Material-efficient microfluidic platform for exploratory studies of visible-light photoredox catalysis. *Angew. Chem., Int. Ed.* **56**, 9847–9850 (2017).

(8) Beulig, R. J., Warias, R., Heiland, J. J., Ohla, S., Zeitler, K. & Belder, D. A droplet-chip/mass spectrometry approach to study organic synthesis at nanoliter scale. *Lab Chip* **17**, 1996–2002 (2017).

(9) Wleklinski, M. *et al*., High-throughput reaction screening using desorption electrospray ionization mass spectrometry. *Chem. Sci.* **9**, 1647–1653 (2018).

(10) Santanilla, A. B. *et al.*, Nanomole-scale high-throughput chemistry for the synthesis of complex molecules. *Science* **347**, 443–448 (2015).

(11) Gesmundo, N. J., Sauvagnat, B., Curran, P. J., Richards, M. P., Andrews, C. L., Dandliker, P. J. & Cernak, T. Nanomole synthesis and affinity ranking. *Nature* **557**, 228–232 (2018).
